# 0s and 1s in marine molecular research: a regional HPC perspective

**DOI:** 10.1093/gigascience/giab053

**Published:** 2021-08-18

**Authors:** Haris Zafeiropoulos, Anastasia Gioti, Stelios Ninidakis, Antonis Potirakis, Savvas Paragkamian, Nelina Angelova, Aglaia Antoniou, Theodoros Danis, Eliza Kaitetzidou, Panagiotis Kasapidis, Jon Bent Kristoffersen, Vasileios Papadogiannis, Christina Pavloudi, Quoc Viet Ha, Jacques Lagnel, Nikos Pattakos, Giorgos Perantinos, Dimitris Sidirokastritis, Panagiotis Vavilis, Georgios Kotoulas, Tereza Manousaki, Elena Sarropoulou, Costas S Tsigenopoulos, Christos Arvanitidis, Antonios Magoulas, Evangelos Pafilis

**Affiliations:** Hellenic Centre for Marine Research, Institute of Marine Biology, Biotechnology and Aquaculture, Former U.S. Base of Gournes, P.O. Box 2214, 71003, Heraklion, Crete, Greece; Department of Biology, University of Crete, Voutes University Campus, P.O. Box 2208, 70013, Heraklion, Crete, Greece; Hellenic Centre for Marine Research, Institute of Marine Biology, Biotechnology and Aquaculture, Former U.S. Base of Gournes, P.O. Box 2214, 71003, Heraklion, Crete, Greece; Hellenic Centre for Marine Research, Institute of Marine Biology, Biotechnology and Aquaculture, Former U.S. Base of Gournes, P.O. Box 2214, 71003, Heraklion, Crete, Greece; Hellenic Centre for Marine Research, Institute of Marine Biology, Biotechnology and Aquaculture, Former U.S. Base of Gournes, P.O. Box 2214, 71003, Heraklion, Crete, Greece; Hellenic Centre for Marine Research, Institute of Marine Biology, Biotechnology and Aquaculture, Former U.S. Base of Gournes, P.O. Box 2214, 71003, Heraklion, Crete, Greece; Department of Biology, University of Crete, Voutes University Campus, P.O. Box 2208, 70013, Heraklion, Crete, Greece; Hellenic Centre for Marine Research, Institute of Marine Biology, Biotechnology and Aquaculture, Former U.S. Base of Gournes, P.O. Box 2214, 71003, Heraklion, Crete, Greece; Hellenic Centre for Marine Research, Institute of Marine Biology, Biotechnology and Aquaculture, Former U.S. Base of Gournes, P.O. Box 2214, 71003, Heraklion, Crete, Greece; Hellenic Centre for Marine Research, Institute of Marine Biology, Biotechnology and Aquaculture, Former U.S. Base of Gournes, P.O. Box 2214, 71003, Heraklion, Crete, Greece; School of Medicine, University of Crete, Voutes University Campus, 70013 Heraklion, Crete, Greece; Hellenic Centre for Marine Research, Institute of Marine Biology, Biotechnology and Aquaculture, Former U.S. Base of Gournes, P.O. Box 2214, 71003, Heraklion, Crete, Greece; Hellenic Centre for Marine Research, Institute of Marine Biology, Biotechnology and Aquaculture, Former U.S. Base of Gournes, P.O. Box 2214, 71003, Heraklion, Crete, Greece; Hellenic Centre for Marine Research, Institute of Marine Biology, Biotechnology and Aquaculture, Former U.S. Base of Gournes, P.O. Box 2214, 71003, Heraklion, Crete, Greece; Hellenic Centre for Marine Research, Institute of Marine Biology, Biotechnology and Aquaculture, Former U.S. Base of Gournes, P.O. Box 2214, 71003, Heraklion, Crete, Greece; Hellenic Centre for Marine Research, Institute of Marine Biology, Biotechnology and Aquaculture, Former U.S. Base of Gournes, P.O. Box 2214, 71003, Heraklion, Crete, Greece; Bull SAS, Rue du Gros Caillou, 78340 Les Clayes-sous-Bois, France; Institut National de Recherche pour l’Agriculture, l’Alimentation et l’Environnement, UR1052, Génétique et Amélioration des Fruits et Légumes, 67 Allée des Chênes, Centre de Recherche Provence-Alpes-Côte d’Azur, Domaine Saint Maurice, CS60094, 84143 Montfavet Cedex, France; Hellenic Centre for Marine Research, Institute of Marine Biology, Biotechnology and Aquaculture, Former U.S. Base of Gournes, P.O. Box 2214, 71003, Heraklion, Crete, Greece; Hellenic Centre for Marine Research, Institute of Marine Biology, Biotechnology and Aquaculture, Former U.S. Base of Gournes, P.O. Box 2214, 71003, Heraklion, Crete, Greece; Hellenic Centre for Marine Research, Network Operation Center, Former U.S. Base of Gournes, P.O. Box 2214, 71003, Heraklion, Crete, Greece; Hellenic Centre for Marine Research, Network Operation Center, Former U.S. Base of Gournes, P.O. Box 2214, 71003, Heraklion, Crete, Greece; Hellenic Centre for Marine Research, Institute of Marine Biology, Biotechnology and Aquaculture, Former U.S. Base of Gournes, P.O. Box 2214, 71003, Heraklion, Crete, Greece; Hellenic Centre for Marine Research, Institute of Marine Biology, Biotechnology and Aquaculture, Former U.S. Base of Gournes, P.O. Box 2214, 71003, Heraklion, Crete, Greece; Hellenic Centre for Marine Research, Institute of Marine Biology, Biotechnology and Aquaculture, Former U.S. Base of Gournes, P.O. Box 2214, 71003, Heraklion, Crete, Greece; Hellenic Centre for Marine Research, Institute of Marine Biology, Biotechnology and Aquaculture, Former U.S. Base of Gournes, P.O. Box 2214, 71003, Heraklion, Crete, Greece; Hellenic Centre for Marine Research, Institute of Marine Biology, Biotechnology and Aquaculture, Former U.S. Base of Gournes, P.O. Box 2214, 71003, Heraklion, Crete, Greece; LifeWatch European Research Infrastructure Consortium, Sector II-III Plaza de España, 41071, Seville, Spain; Hellenic Centre for Marine Research, Institute of Marine Biology, Biotechnology and Aquaculture, Former U.S. Base of Gournes, P.O. Box 2214, 71003, Heraklion, Crete, Greece; Hellenic Centre for Marine Research, Institute of Marine Biology, Biotechnology and Aquaculture, Former U.S. Base of Gournes, P.O. Box 2214, 71003, Heraklion, Crete, Greece

**Keywords:** marine research, high performance computing, containerization, computational requirements, high-throughput sequencing, research infrastructures, biodiversity, biotechnology, aquaculture

## Abstract

High-performance computing (HPC) systems have become indispensable for modern marine research, providing support to an increasing number and diversity of users. Pairing with the impetus offered by high-throughput methods to key areas such as non-model organism studies, their operation continuously evolves to meet the corresponding computational challenges. Here, we present a Tier 2 (regional) HPC facility, operating for over a decade at the Institute of Marine Biology, Biotechnology, and Aquaculture of the Hellenic Centre for Marine Research in Greece. Strategic choices made in design and upgrades aimed to strike a balance between depth (the need for a few high-memory nodes) and breadth (a number of slimmer nodes), as dictated by the idiosyncrasy of the supported research. Qualitative computational requirement analysis of the latter revealed the diversity of marine fields, methods, and approaches adopted to translate data into knowledge. In addition, hardware and software architectures, usage statistics, policy, and user management aspects of the facility are presented. Drawing upon the last decade’s experience from the different levels of operation of the Institute of Marine Biology, Biotechnology, and Aquaculture HPC facility, a number of lessons are presented; these have contributed to the facility’s future directions in light of emerging distribution technologies (e.g., containers) and Research Infrastructure evolution. In combination with detailed knowledge of the facility usage and its upcoming upgrade, future collaborations in marine research and beyond are envisioned.

## Background

The ubiquitous marine environments (more than 70% of the global surface [1]) mold Earth’s conditions to a great extent. The interconnected abiotic [2] and biotic factors (from bacteria [2] to megafauna [3]), shape biogeochemical cycles [4] and climates [5, 6] from local to global scales. In addition, marine systems have high socio-economic value [7] as an essential source of food and by supporting renewable energy and transport, among other services [8]. The study of marine environments involves a series of disciplines (scientific fields): from Biodiversity [9] and Oceanography to (eco)systems biology [10] and from Biotechnology [11] to Aquaculture [12].

To shed light on the evolutionary history of (commercially important) marine species [13], as well as on how invasive species respond and adapt to novel environments [14], the analysis of their genetic stock structure is fundamental [15]. Similarly, biodiversity assessment is essential to elucidate ecosystem functioning [16] and to identify taxa with potential for bioprospecting applications [17]. Furthermore, systems biology approaches provide both theoretical and technical backgrounds in which integrative analyses flourish [18]. However, conventional methods do not offer the information needed to explore the aforementioned scientific topics.

High-throughput sequencing (HTS) and sister methods have launched a new era in many biological disciplines [19, 20]. These technologies allowed access to the genetic, transcript, protein, and metabolite repertoire [21] of studied taxa or populations, and facilitated the analysis of organism-environment interactions in communities and ecosystems [22]. Whole-genome sequencing and whole-transcriptome sequencing approaches provide valuable information for the study of non-model taxa [23]. This information can be further enriched by genotyping-by-sequencing approaches, such as restriction site–associated DNA sequencing [24], or by investigating gene expression dynamics through Differential Expression (DE) analyses [25]. Moving from single species to assemblages, molecular-based identification and functional profiling of communities has become available through marker (metabarcoding), genome (metagenomics), or transcriptome (metatranscriptomics) sequencing from environmental samples [26]. To a great extent, these methods address the problem of how to produce and get access to the information on different biological systems and molecules.

These 0s and 1s of information (i.e., the data) come along with challenges regarding their management, analysis, and integration [27]. The computational requirements for these tasks exceed the capacity of a standard laptop/desktop by far, owing to the sheer volume of the data and to the computational complexity of the bioinformatic algorithms employed for their analysis. For example, building the *de novo* genome assembly of a non-model Eukaryote may require algorithms of nondeterministic polynomial time complexity. This analysis can reach up to several hundreds or thousands of GB of memory (RAM) [28]. Hence, the challenges of how to exploit all these data and how to transform data into knowledge set the present framework in biological research [29, 30].

To address these computational challenges, the use of high-performance computing (HPC) systems has become essential in life sciences and systems biology [31]. HPC is the scientific field that aims at the optimal incorporation of technology, methodology, and the application thereof to achieve “the greatest computing capability possible at any point in time and technology" [32]. Such systems range from a small number to several thousands of interconnected computers (compute nodes). According to the Partnership for Advanced Computing in Europe, the European HPC facilities are categorized as: (i) European Centres (Tier 0), (ii) national centers (Tier 1), and (iii) regional centers (Tier 2) [33]. As the Partnership for Advanced Computing in Europe highlights, “computing drives science and science drives computing" in a great range of scientific fields, from the endeavor to maintain a sustainable Earth to efforts to expand the frontiers in our understanding of the universe [34]. On top of the heavy computational requirements, biological analyses come with a series of other practical issues that often affect the bioinformatics-oriented HPC systems.

Researchers with purely biological backgrounds often lack the coding skills or even the familiarity required for working with Command Line Interfaces [34]. Virtual Research Environments are web-based e-service platforms that are particularly useful for researchers lacking expertise and/or computing resources [35]. Another common issue is that most analyses include a great number of steps, with the software used in each of these having equally numerous dependencies. A lack of continuous support for tools with different dependencies, as well as frequent and non-periodical versioning of the latter, often results in broken links and further compromises the reproducibility of analyses [36]. Widely used containerization technologies—e.g., Docker [37] and Singularity [38]—ensure reproducibility of software and replication of the analysis, thus partially addressing these challenges. By encapsulating software code along with all its corresponding dependencies in such containers, software packages become reproducible in any operating system in an easy-to-download-and-install fashion, on any infrastructure.

The Institute of Marine Biology Biotechnology and Aqua-culture (IMBBC) has been developing a computing hub that, in conjunction with national and European Research Infrastructures (RIs), can support state-of-the-art marine research. The regional IMBBC HPC facility allows processing of data that derive from the Institute’s sequencing platforms and expeditions and from multiple external sources in the context of interdisciplinary studies. Here, we present insights from a thorough analysis of the research supported by the facility and some of its latest usage statistics in terms of resource requirements, computational methods, and data types; the above have contributed in shaping the facility along its lifespan.

## The IMBBC HPC Facility: From a Single Server to a Tier 2 System

**Figure 1:**
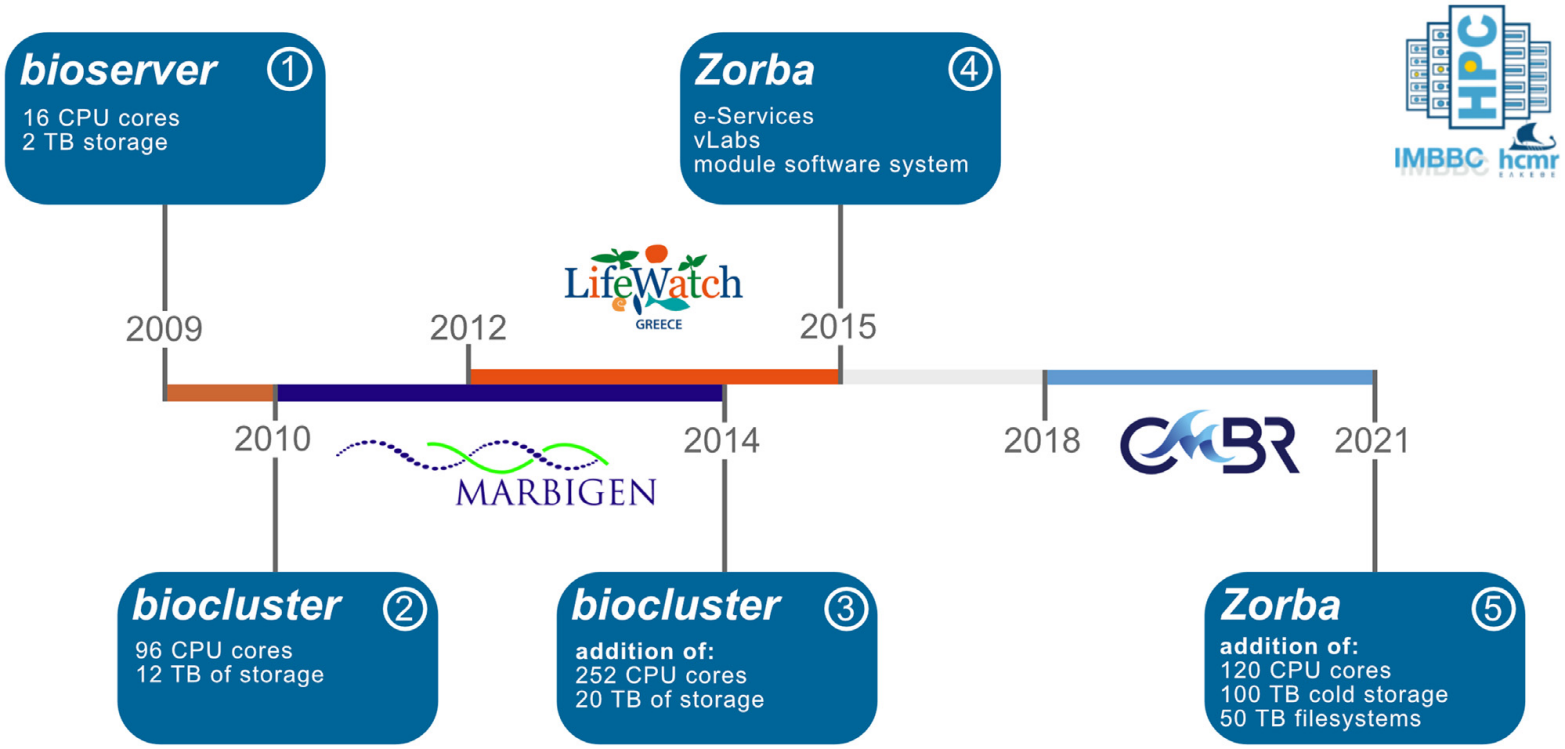
Evolution of the IMBBC HPC facility during the past 12 years, with hardware upgrades (blue boxes) and funding milestones (logos of RIs) highlighted. A single server that launched the bioinformatics era in 2009 evolved to the current Tier 2 system *Zorba* (Box 4), which allows processing of a wide variety of information from DNA sequences to biodiversity data. Different names of the facility denote distinct system architectures.

The IMBBC HPC facility was launched in 2009 to support computational needs over a range of scientific fields in marine biology, with a focus on non-model taxa [39]. The facility was initiated as an infrastructure of the Institute of Marine Biology and Genetics of the Hellenic Centre for Marine Research. Its development has followed the development of national RIs (Fig. 1; also see Section A1 in Zafeiropoulos et al. [40]). The first nodes were used to support the analysis of data sets generated from methods such as eDNA metabarcoding and multiple omics. Since 2015, the facility also supports Virtual Research Environments, including e-services and virtual laboratories. The current configuration of the facility presented herein is named *Zorba* (Fig. 1, Box 4) and will be upgraded within 2021 (see Section Future Directions). Hereafter, *Zorba* refers to the specific system setup from 2015 and onwards, while the facility throughout its lifespan will be referred to as “IMBBC HPC".

**Figure 2:**
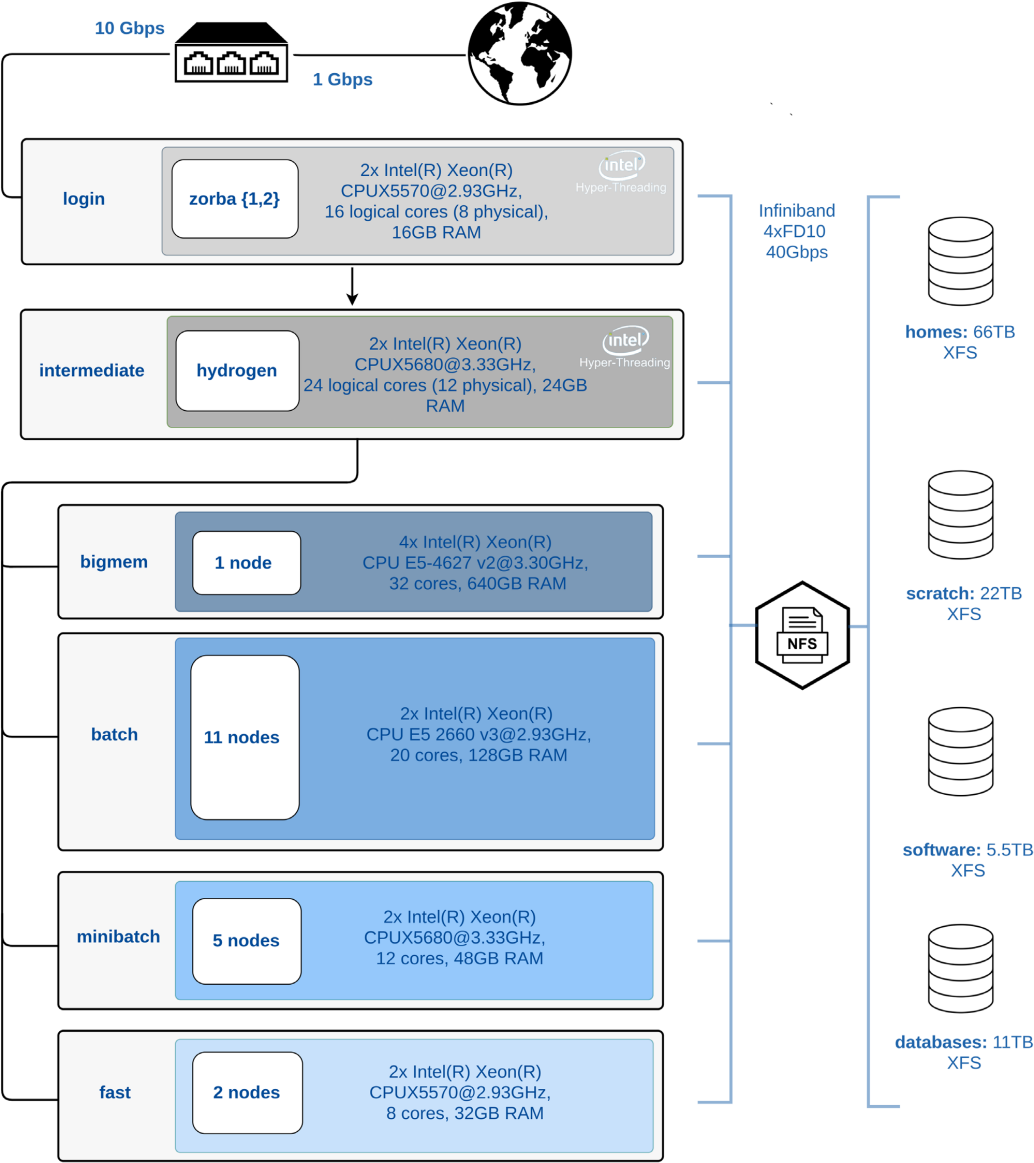
Block diagram of the *Zorba* architecture. This is the IMBBC HPC facility architecture in its current setup, after 12 years of development. There are 2 login nodes and 1 intermediate where users may develop their analyses. Computational nodes are split into 4 partitions with different specs and policy terms: bigmem supporting processes requiring up to 640 GB RAM, batch handling mostly (but not exclusively) parallel-driven jobs (either in a single node or across several nodes), minibatch aiming to serve parallel jobs with reduced resource requirements, and fast partition for non-intensive jobs. All servers, except file systems, run Debian 9 (kernel 4.9.0-8-amd64). CCBY icons from the Noun Project: “nfs file document icon" by IYIKON, PK; “Earth" By mungang kim, KR; “database": By Vectorstall, PK; “switch" by Bonegolem, IT

*Zorba* currently consists of 328 CPU cores, 2.3 TB total memory, and 105 TB storage. Job submission takes place on the 4 available computing partitions, or queues, as explained in Fig. 2. *Zorba* at its current state achieves a peak performance of 8.3 trillion double-precision floating-point operations per second, or 8.3 Tflops, as estimated by LinPack benchmarking [41]. On top of these, a total 7.5 TB is distributed to all servers for the storage of environment and system files. Interconnection of both the compute and login nodes takes place via an infiniband interface with a capacity of 40 Gbps, which features very high throughput and very low latency. Infiniband is also used for a switched interconnection between the servers and the 4 available file systems. A thorough technical description of *Zorba* is available in Section A2 of Zafeiropoulos et al. [40].

More than 200 software packages are currently installed and available to users at *Zorba*, covering the most common analysis types. These tools allow assembly, HTS data preprocessing, phylogenetic tree construction, ortholog finding, and population structure modeling, to name a few. Access to these packages is provided through Environment Modules, a broadly used means of accessing software in HPC systems [42].

During the last 2 years, *Zorba* has been moving from system-dependent pipelines previously developed at IMBBC (e.g., ParaMetabarCoding) towards containerization of available and new pipelines/tools. A complete metabarcoding analysis tool for various marker genes (PEMA) [43], the chained and automated use of STACKS, software for population genetics analysis from short-length sequences [44] (latest version), a set of statistical functions in R for the computation of biodiversity indices, and analyses in cases of high computational demands [45], as well as a programming workflow for the automation of biodiversity historical data curation (DECO) are among the in-house developed containers. The standard container/image format used on *Zorba* is Singularity. Singularity images can be served by any *Zorba* partition; Docker images can run instantly as Singularity images. A thorough description of the software containers developed in *Zorba* can be found in Section D of Zafeiropoulos et al. [40].

*Zorba*'s daily functioning is ensured by a core team of 4 full-time, experienced staff: a hardware officer, 2 system administrators, and a permanent researcher in biodiversity informatics and data science.

More than 70 users (internal and external scientists), investigators, postdoctoral researchers, technicians, and doctoral/postgraduate students have gained access to the HPC infrastructure thus far. Support is provided officially through a help desk ticketing system. An average of 31 requests/month have been received (since June 2019), with the most demanded categories being troubleshooting (38.2%) and software installation (23.8%). Since October 2017, monthly meetings among HPC users have been established to regularly discuss such issues.

Proper scheduling of the submitted jobs and fair resource sharing is a major task that needs to be confronted day to day. To address this, a specific usage policy for each of the various partitions and a scheduling software tool set have been adopted in *Zorba*. Policy terms are dynamically adapted to the HPC hardware architecture and to the usage statistics, with revisions being discussed between the HPC core team and users. The Simple Linux Utility for Resource Management (SLURM) open-source cluster management system orchestrates the job scheduling and allocates resources, and a booking system helps users to organize their projects and administrators to monitor the resource reservations on a mid- to long-term basis. A SLURM Database Daemon (slurmdbd) has also been installed to allow logging and recording of job usage statistics into a separate SQL database (see Section C1 in Zafeiropoulos et al. [40]). An extended description of user and job administrations and orchestration can be found in Section C1 of Zafeiropoulos et al. [40]).

Training has been an integral component of the HPC facility mindset since its launch and enables knowledge sharing across MSc and PhD students and researchers within and outside the Institute. Introductory courses are organized on a regular basis, aimed at familiarizing new users with Unix environments, programming, and HPC usage policy and resource allocation (e.g., job submission in SLURM). Furthermore, the IMBBC HPC facility has served, since 2011, as an international training platform for specific types of bioinformatic analyses (see Section C2 in Zafeiropoulos et al. [40]). For instance, the facility has provided computational resources for workshops on Microbial Diversity, Genomics and Metagenomics, Genomics in Biodiversity, Next-Generation Sequencing technologies and informatics tools for studying marine biodiversity and adaptation in the long term, or Ecological Data Analysis using R. The plan is to enhance and diversify the educational component of the HPC facility by providing courses on a more permanent basis and targeting a larger audience. An extensive listing of training activities is given in Section C2 of Zafeiropoulos et al. [40].

## Computational Breakdown of the IMBBC HPC-Supported Research

Systematic labelling of IMBBC HPC-supported published studies (*n* = 47) was performed to highlight their resource requirements. Each study was manually labelled with the relevant scientific field, the data acquisition method, the computational methods, and its resource requirements; all the annotations were validated by the corresponding authors (see Section D2 in Zafeiropoulos et al. [40]). It should be stated that the conclusions of this overview are specific to the studies conducted at IMBBC.

**Figure 3:**
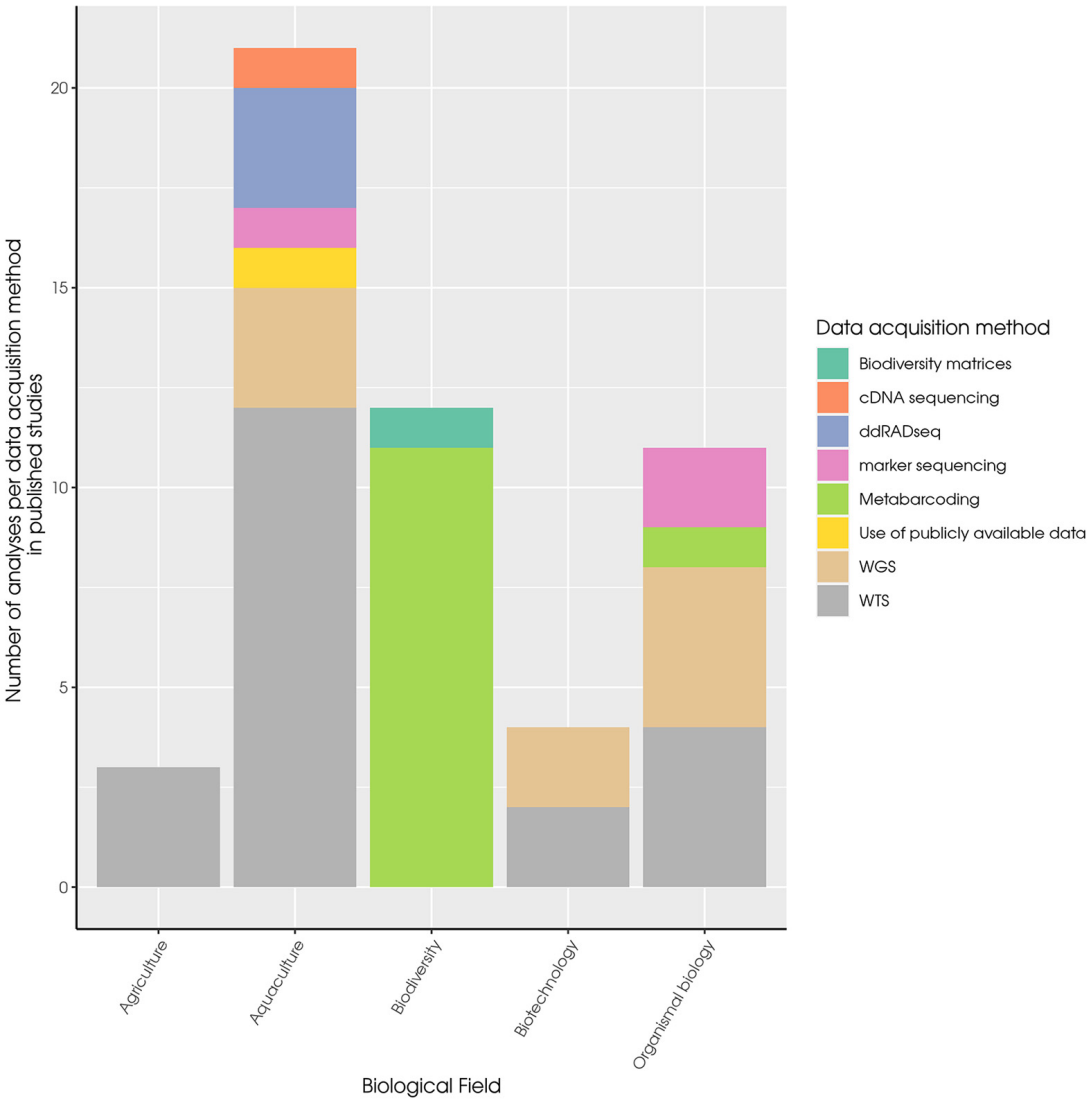
Bar chart with the number of publications that have used IMBBC HPC facility resources, grouped by scientific field. The different methods for data acquisition are also presented. WGS, whole-genome sequencing; WTS, whole-transcriptome sequencing.

The scientific fields of Aquaculture (~40% of studies), Biodiversity (~26% of studies), and Organismal biology (~19% of studies) account for the majority of the research publications supported by the IMBBC HPC facility (Fig. 3; Supplementary File imbbc_hpc_labelling_data.xlsx in Zafeiropoulos et al. [40]).

In comparison, studies in the Biotechnology and Agriculture fields indicate contemporary and beyond-marine orientations of research at IMBBC, respectively (see Section B2 in Zafeiropoulos et al. [40]). In addition, 8 methods of data acquisition (experimental or in silico) have been defined (Fig. 3). Among these methods, whole-genome sequencing and whole-transcriptome sequencing have been widely used in multiple fields (Biotechnology, Organismal Biology, Aquaculture). Conversely, Double digest restriction-site associated sequencing (ddRADseq) has been solely employed for population genetic studies in the context of Aquaculture.

**Figure 4:**
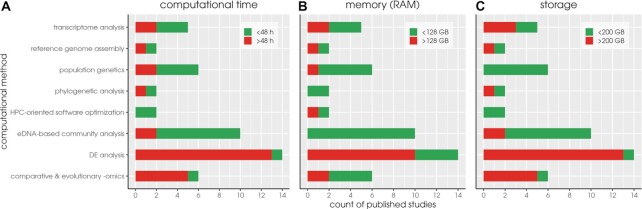
Red bars denote published research with high resource requirements of the various computational methods employed at the IMBBC HPC facility due to (a) long computational times (>48 h), (b) high memory requirements (>128 GB), or (c) high storage requirements (>200 GB). For instance, no eDNA-based community analyses performed at *Zorba* thus far have required a large amounts of memory.

The 47 published studies employed different computational methods (sets of tasks executed on the HPC facility). These studies served different purposes, from a range of bioinformatics analyses to HPC-oriented software optimization. The computational methods were categorized in 8 classes (Fig. 4). The resource requirements of each computational method were evaluated in terms of memory usage, computational time, and storage. Reflecting the current *Zorba* capacity, studies which, in any part of their analysis, exceeded 128 GB of memory or/and 48 hours of running time or/and 200 GB physical space were classified as studies with high demands (see Supplementary file imbbc_hpc_labelling_data.xlsx in Zafeiropoulos et al. [40]).

As shown in Fig. 4, the 2 most commonly used computational methods have rather different resource requirements. While DE analysis shows a notable trend for both long computational times (Fig. 4a) and high memory (Fig. 4b), eDNA-based community analysis does not have high resource requirements either in computation time or memory. High memory was commonly associated with computational methods, including *de novo* assembly; all relevant research concerned non-model taxa and involved short-read sequencing or combinations of short- and long-read sequencing. By contrast, phylogenetic analysis studies did not involve intensive RAM use; this is largely due to the fact that software used by IMBBC users adopts parallel solutions for tree construction. Long computational times (Fig. 4a) were most often observed at the functional annotation step in transcriptome analysis, DE analysis, and comparative and evolutionary omics, when this step involved BLAST queries of thousands of predicted genes against large databases, such as nr (NCBI). Finally, a common challenge emerging from all bioinformatic approaches is significant storage limitations (Fig. 4c); this challenge was associated with the use of HTS technologies that produce large amounts of raw data, the analysis of which involves the creation of numerous intermediate files.

Overall, published studies using the IMBBC HPC facility show a degree of variance with respect to the types of tools used (depending on the user, their bioinformatic literacy, and other factors), each of which is more or less optimized with respect to HPC use. Moreover, the variance in computational needs observed within each type of computational method reflects the diversity of the studied taxonomic groups. For instance, transcriptome analysis (involving *de novo* assembly and functional annotation steps) was employed for the study of taxa as diverse as bacteria, sponges, fungi, fish, and goose barnacles. The complexity of each of these organisms’ transcriptomes can, to a large extent, explain the differences observed in computational time, memory, and storage.

Furthermore, *Zorba* CPU and RAM statistics collected since 2019 displayed some overall patterns, including an average computation load per month of less than or close to 50% of its max capacity (50% of 236 kilocorehours/month) for most (20) of the 24 months of the logging period. Memory requirements were also heterogeneous: most (90%) of the 44,000 jobs performed in the same 24-month period required less than 10 GB of RAM, and 0.30% of the jobs required more than 128 GB of RAM (i.e., exceeding the memory capacity of the main compute nodes [batch partition]). The detailed usage statistics of *Zorba* are described in Section B1 and Supplementary file zorba_usage_statistics.xlsx of Zafeiropoulos et al. [40].

## Scientific Impact Stories

Below, some examples of research results that were made possible with the IMBBC HPC facility are described. This list of use cases is by no means exhaustive, but rather an attempt to highlight different fields of research supported by the facility, along with their distinct computational features.

### Invasive species range expansion detected with eDNA data from Autonomous Reef Monitoring Structures

The Mediterranean biodiversity and ecosystems are experiencing profound transformations owing to Lessepsian migration, international shipping, and aquaculture, which lead to the migration of nearly 1,000 alien species [46]. The first step towards addressing the effects of these invasions is monitoring of the introduced taxa. A powerful tool in this direction has been eDNA metabarcoding, which has enchanced detection of invasive species [47], often preceding macroscopic detection. One such example is the first record of the nudibranch *Anteaeolidiella lurana* (Ev. Marcus & Er. Marcus, 1967) in Greek waters in 2020 [48]. An eDNA metabarcoding analysis allowed for detection of the species with high confidence on fouling communities developed on Autonomous Reef Monitoring Structures (ARMS). This finding, confirmed with image analysis of photographic records on a later deployment period, is an example of work conducted within the framework of the European ASSEMBLE plus programme ARMS-MBON (Marine Biodiversity Observation Network). PEMA software [43] was used in this study, as well as in the 30-month pilot phase of ARMS-MBON [49].

### Providing omics resources for large genome-size, non-model taxa

*Zorba* has been used for building and annotating numerous *de novo* genome and transcriptome assemblies of marine species, such as the gilthead sea bream *Sparus aurata* [50] or the greater amberjack *Seriola dumerili* [51]. Both genome and transcriptome assemblies of species with large genomes often exceed the maximum available memory limit, eventually affecting the strategic choices for *Zorba* future upgrades (see Section Future Directions). For instance, building the draft genome assembly of the seagrass *Halophila stipulacea* (estimated genome size 3.5 GB) using Illumina short reads has been challenging even for seemingly simple tasks, such as a kmer analysis [52]. Taking advantage of short- and long-read sequencing technologies to construct high-quality reference genomes, the near-chromosome level genome assembly of *Lagocephalus sceleratus* (Gmelin, 1789) was recently completed as a case study of high ecological interest due to the species’ successful invasion throughout the Eastern Mediterranean [53]. In the context of this study, an automated containerized pipeline allowing high-quality genome assemblies from Oxford Nanopore and Illumina data was developed (SnakeCube [54]). The availability of standardized pipelines offers great perspective for in-depth studies of numerous marine species of interest in aquaculture and conservation biology, including rigorous phylogenomic analyses to position each species in the tree of life (e.g., Natsidis et al. [55]).

### DE analysis of aquaculture fish species sheds light on critical phenotypes

Distinct, observable properties, such as morphology, development, and behavior, characterize living taxa. The corresponding phenotypes may be controlled by the interplay between specific genotypes and the environment. To capture an individual’s genotype at a specific time point, molecular tools for transcript quantification have followed the fast development of technologies, with Expressed Sequence Tags as the first approach to be historically used, especially suited for non-model taxa [56]. Nowadays, the physiological state of aquaculture species is retrieved through investigation of stage-specific and immune- and stress response–specific transcriptomic profiles using RNAseq. The corresponding computational workflows involve installing various tools at *Zorba* and implementing a series of steps that often take days to compute. These analyses, besides detecting transcripts at a specific physiological state, have successfully identified regulatory elements, such as microRNAs. Through the construction of a regulatory network with putative target genes, microRNAs have been linked to the transcriptome expression patterns. The most recent example is the identification of microRNAs and their putative target genes involved in ovary maturation [57].

### Large-scale ecological statistics: are all taxa equal?

The nomenclature of living organisms, as well as their descriptions and their classifications under a specific nomenclature code, have been studied for more than 2 centuries. Up to now, all the species present in an ecosystem have been considered equal in terms of their contributions to diversity. However, this axiom has been tested only once before, on the United Kingdom’s marine animal phyla, showing the inconsistency of the traditional Linnaean classification between different major groups [58]. In Arvanitidis et al. [59], the average taxonomic distinctness index (Δ+) and its variation (*Lambda*+) were calculated on a matrix deriving from the complete World Register of Marine Species [60], containing more than 250,000 described species of marine animals. It is the R-vLab web application, along with its HPC high RAM back-end components (on bigmem, see Section The IMBBC HPC Facility: From a Single Server to a Tier 2 System) that made such a calculation possible. This is the first time such a hypothesis has been tested on a global scale. Preliminary results show that the 2 biodiversity indices exhibit complementary patterns and that there is a highly significant yet non-linear relationship between the number of species within a phylum and the average distance through the taxonomic hierarchy.

### Discovery of novel enzymes for bioremediation

Polychlorinated biphenyls are complex, recalcitrant pollutants that pose a serious threat to wildlife and human health. The identification of novel enzymes that can degrade such organic pollutants is being intensively studied in the emerging field of bioremediation. In the context of the Horizon 2020 Tools And Strategies to access original bioactive compounds by Cultivating MARine invertebrates and associated symbionts (TASCMAR) project, global ocean sampling provided a large biobank of fungal invertebrate symbionts and, through large-scale screening and bioreactor culturing, a marine-derived fungus able to remove a polychlorinated biphenyl compound was identified for the first time. *Zorba* resources and domain expertise in fungal genomics were used as a Centre for the Study and Sustainable Exploitation of Marine Biological Resources (CMBR) service for the analysis of multi-omic data for this symbiont. Following genome assembly of *Cladosporium sp*. TM-S3 [61], transcriptome assembly and a phylogenetic analysis revealed the full diversity of the symbiont’s multicopper oxidases, enzymes commonly involved in oxidative degradation [62]. Among these, 2 laccase-like proteins shown to remove up to 71% of the polychlorinated biphenyl compound are now being expressed to optimize their use as novel biocatalysts. This step would not have been possible without the annotation of the *Cladosporium* genome with transcriptome data; mapping of the purified enzymes’ LC-MS (Liquid chromatography–mass spectrometry) spectra against the set of predicted proteins allowed for identification of their corresponding sequences.

## Lessons Learned

### Depth and breadth are both required for a bioinformatics-oriented HPC

In our experience, the vast majority of the analyses run at the IMBBC HPC infrastructure are CPU-intensive. RAM-intensive jobs (>128 GB RAM, see Section Computational Breakdown of the IMBBC HPC-Supported Research) represent only ~0.3% of the total jobs executed over the last 2 years (see Section B1 in Zafeiropoulos et al. [40]). Despite the difference in the frequency of executed jobs with distinct requirements, serving both types of jobs and ensuring their successful completion is equally important for addressing fundamental marine research questions (as shown in Section Computational Breakdown of the IMBBC HPC-Supported Research). The need for both HPC depth (a few high-memory nodes) and breadth (a number of slimmer nodes) has been previously reported [31]. This need reflects the idiosyncrasy of different bioinformatics analysis steps, often even within the same workflow. High-memory nodes are necessary for tasks such as *de novo* assembly of large genomes, while the availability of as many less powerful nodes as possible can speed up the execution of less demanding tasks and free resources for other users. Future research directions and the available budget further dictate tailoring of the HPC depth and breadth. Cloud-based services—e.g., for containerized workflows—may also facilitate this process once these become more affordable.

### Quota … overloaded

We observed that independently of the type of analysis, storage was an issue for all *Zorba* users (Fig. 4). A high percentage of these issues relate to the raw data from HTS projects. These data are permanently stored in the home directories, occupying significant space. This, in conjunction with the fact that users delete their data with great reluctance, makes storage a major issue of daily use in *Zorba*. In specific cases where users’ quota was exceeded uncontrollably, the *Zorba* team has been applying compression of raw and output data in contact with the user, but this is by no means a stable strategy. More generally, with the performance of the existing storage configuration in *Zorba* close to reaching its limits due to the increase in users and its concurrent use, several solutions have been adopted to resolve the issue. The most long-lasting solution has been the adoption of a per user quota system to allow storage sustainability and fairness in our allocation policy. This quota system nevertheless constitutes a limiting factor in pipeline execution, since lots of software tools produce unpredictably too many intermediate files, which not only increase storage but also cause job failures due to space restrictions. We managed the above issue by adding a scratch file system as an intermediate storage area for the runtime capacity needs. Following completion of their analysis, a user retains only the useful files and the rest are permanently removed. A storage upgrade scheduled within 2021 (see Section Future Directions) is expected to alleviate current storage challenges in *Zorba*. However, given the ever-increasing data production (e.g., as the result of decreasing sequencing costs and/or of rising imaging technologies), the responsible storage use approaches described here remain only partial solutions to anticipated future storage needs. Centralized (Tier 1 or higher) storage solutions represent a longer-term solution, which is in line with current views on how to handle big data generated by international research consortia in a long-lasting manner.

### Continuous intercommunication among different disciplines matters

Smooth functioning of an HPC system and exploitation of its full potential for research requires stable employment of a core team of computer scientists and engineers, in close collaboration with an extended team of researchers. At least 4 disciplines are involved in *Zorba*-related issues: computer scientists, engineers, biologists (in the broad sense, including ecologists, genomicists, etc.), and bioinformaticians with varying degrees of literacy in biology and informatics and various domain specializations (comparative genomics, biodiversity informatics, bacterial metagenomics, etc). The continuous communication among representatives of these 4 disciplines has substantially contributed to research supported by *Zorba* and to the evolution of the HPC system itself over time. In our experience, an HPC system cannot function effectively and for long without full-time system administrators, nor with bioinformaticians alone. Although it has not been the case since the system’s onset, investment in monthly meetings, seminars, and training events (in biology, containers, domain-specific applications, and computer science; see Section The IMBBC HPC Facility: From a Single Server to a Tier 2 System) is the only way to establish stable intercommunication among different players of an HPC system. Such proximity translates into timely and adequate systems and bioinformatics analysis support, an element that in its turn translates into successful research (see Section Computational Breakdown of the IMBBC HPC-Supported Research). It should be noted that the overall good experience in connectivity among different HPC players derives from *Zorba* being a Tier 2 system, with a number of active permanent users in double digits. The establishment of such inter-communication was relatively straightforward to implement with periodic meetings and the assistance of ticketing and other management solutions (see Section C1 in Zafeiropoulos et al. [40]).

### The way forward: develop locally and share and deploy centrally

The various approaches regarding the function of an HPC system are strongly related to the different viewpoints of the academic communities towards the relatively new disciplines of bioinformatics and big data. These approaches are strongly affected by national and international decisions that affect the ability to fund supercomputer systems. There are advantages in deploying bioinformatics-oriented HPC systems in centralized (Tier 0 and Tier 1) facilities: better prices at hardware purchases, easier access to HPC-tailored facilities (for instance, in terms of the cooling system and physical space), or experienced technical personnel (see also Lampa et al. [31]). However, synergies between regional (Tier 2) and centralized HPC systems are fundamental for moving forward in supporting the diverse and demanding needs of bioinformatics. An example of such synergies concerns technical solutions (e.g., containerization) that address long-standing software sharing issues. In our experience, a workflow/pipeline can be developed by experts within the context of a specific project in a regional HPC facility. Once a production version of the pipeline is packaged, it can be distributed to centralized systems to cover a broader user audience (see Section The IMBBC HPC Facility From a Single Server to a Tier 2 System). Singularity containers have been developed to utterly suit HPC environments, mostly because they permit root access of the system in all cases. In addition, Singularity is compatible with all Docker images and can be used with Graphics Processing Units (GPUs) and Message Passing Interface (MPI) applications. This is why we chose to run containers in a Singularity format at *Zorba*. However, as Docker containers are widely used, especially in cloud computing (see more about cloud computing in Section Cloud Computing), workflows and services produced at IMBBC are offered in both container formats. Containers are an already established technology, used by the biggest cloud providers worldwide and increasingly by non-profit research institutes. Despite indirect costs (e.g., costs to containerize legacy software), we believe that these technologies will become the norm in the future, especially in the context of reproducibility and interoperability of bioinformatics analysis.

### Software optimizations for parallel execution

The most common ways of achieving implicit or explicit parallelization in modern multicore systems for bioinformatics, computational biology, and systems biology software tools are the software threads—provided by programming languages—and/or the OpenMP API [63]. These types of multiprocessing make good use of the available cores on a multicore system (single node), but they are not capable of combining the available CPU cores from more than 1 node. Some other software tools use MPI to spawn processing chunks to many servers and/or cores or (even better) combine MPI with OpenMP/Threads to maximize the parallelization in hybrid models of concurrency. Such designs are now used to a great extent in some cases, such as phylogeny inference software that makes use of Monte Carlo Markov Chain samplers. However, these cases are but a small number compared to the majority of bioinformatics tasks, while their usage in other analyses is low. At the hardware level, simultaneous multithreading is not enabled in the compute nodes of the IMBBC HPC infrastructure. Since the majority of analyses running on the cluster demand dedicated cores, hardware multithreading does not perform well. In our experience, the existence of more (logical) cores in compute nodes misleads the least experienced users into using more threads than the physically available ones, which slows down their executions. In comparison, assisting servers (filesystems, login nodes, web servers) make use of hardware multithreading, since they serve numerous small tasks from different users/sources that commonly contain Input/Output (I/O) operations. GPUs provide an alternative way for parallel execution, but they are supported by a limited number of bioinformatics software tools. Nevertheless, GPUs can optimize the execution process in specific, widely used bioinformatic analyses, such as sequence alignment [64, 65], image processing in microtomography (e.g., microCT), or basecalling of Nanopore raw data.

## Cloud Computing

A recent alternative to traditional HPC systems, such as that described in this review, is cloud computing. Cloud computing is the way of organizing computing resources so they are provided over the Internet (“the cloud"). This paradigm of computing requires the minimum management effort possible [66]. Cloud computing providers exist in both commercial vendors and academic/publicly funded institutions and infrastructures (for more on cloud computing for bioinformatics, see Langmead and Nellore [67]). Computing resources can be reserved from individuals, institutions, organizations, or even scientific communities. The most widely-known commercial cloud providers are the “big 3” of cloud computing—namely, Amazon Web Services, Google Cloud Platform, and Microsoft Azure—while other cloud vendors are constantly emerging. Academic/publicly funded providers are also available: e.g., the EMBL–EBI Embassy Cloud.

Cloud computing services are being increasingly adopted in research, mainly because they offer simplicity and high availability to users with reduced or even no experience in HPC systems, through web interfaces. For this type of user, the time needed for data manipulation, software installation, and user-system interaction is significantly reduced compared to using a local HPC facility.

Container technologies, especially Docker, along with container-management systems such as Kubernetes combined with OpenStack, have been widely used in a number cloud computing systems, in particular in the research domain. It should be noted, however, that tool experimentation and benchmarking is more limited in cloud computing compared to local facilities and is costly, since it demands additional core hours of segmented computation. In-house HPC infrastructures can be fully configured to suit specific research area needs (storage available, fast interconnection for MPI jobs, number of CPUs versus available RAM, assisting services, etc.). Moreover, in cases where InfiniBand interconnection, a computer networking communications standard, is adopted in HPC, the performance in jobs and software that take advantage of it is substantial. Given the features and advantages of each approach (mentioned above) one could foresee the scenario of combining them to address the research community needs.

## Future Directions

An upgrade of the existing hardware design of *Zorba* has been scheduled in 2021, funded by the CMBR research infrastructure (Fig. 1). More specifically:

- 3 nodes of 40 CPU physical cores will be added through new partitions (120 cores in total);
- the total RAM will be increased by 3.5 TB;
- 100 TB of cold storage will be installed and is expected to alleviate the archiving problem at the existing homes/scratch file systems; and
- the total usable existing storage capacity for users in home and scratch partitions will be increased by approximately 100 TB.

With this upgrade, it is expected that the total computational power of *Zorba* will be increased by approximately 6 TFlops, while the infrastructure will be capable of serving memory-intensive jobs requiring up to 1.5 TB of RAM, hosted on a single node. Eventually, more users will be able to concurrently load and analyze big data sets on the file systems. Over the coming 2 years, *Zorba* is also expected to have 2 major additions:

- the acquisition of a number of GPU nodes to build a new partition, especially for serving software that has been ported to run on GPUs; and
- the design of a parallel file system (Ceph or Lustre) to optimize concurrent I/O operations to speed up CPU-intensive jobs.

The expectation is that the upcoming upgrade of *Zorba* will further enhance collaborations with external users, since the types of bioinformatic tasks supported by the infrastructure are common to other disciplines beyond marine science, such as environmental omics research in the broad term. A nationwide survey targeting the community of researchers studying the environment and adopting the same approaches (HTS, biodiversity monitoring) has revealed that their computational and training needs are on the rise (A. Gioti et al., unpublished observations). Usage peaks and valleys were observed in *Zorba* (see Section B1 in Zafeiropoulos et al. [40]), similarly to other HTS-oriented HPC systems [31]. It is therefore feasible to share *Zorba*'s idling time with other scientific communities. Besides, the *Zorba* upgrade is very timely in coming during a period where additional computational infrastructures emerge: the Cloud infrastructure Hypatia, funded by the Greek node of ELIXIR, is entering its production phase. It will constitute a national Tier 1 HPC facility, designed to host ~50 computational nodes of different capabilities (regular servers, GPU-enabled servers, Solid-State Drive-enabled servers, etc.) and provide users the option to either create custom virtual machines for their computational services or to upload and execute workflows of containerized scientific software packages. In this context, a strategic combination of *Zorba* and *Hypatia* is expected to contribute to a strong computational basis in Greece. It is also expected that *Zorba* functionality will be augmented also through its connection with the Super Computing Installations of LifeWatch ERIC (European Research Infrastructure Consortium) (e.g., Picasso facility in Malaga, Spain). Building upon the lessons learned in the last 12 years, a foreseeable challenge for the facility is the enhancement of its usage monitoring to the example of international HPC systems [68], in order to allow even more efficient use of computational resources.

## Conclusions

*Zorba* is an established Tier 2 HPC regional facility operating in Crete, Greece. It serves as an interdisciplinary computing hub in the eastern Mediterranean, where studies in marine conservation, invasive species, extreme environments, and aquaculture are of great scientific and socio-economic interest. The facility has supported, since its launch over a decade ago, a number of different fields of marine research, covering all kingdoms of life; it can also share part of its resources to support research beyond the marine sciences.

The operational structure of *Zorba* enables continuous communication between users and administrators for more effective user support, troubleshooting, and job scheduling. More specifically, training, regular meetings, and containerization of in-house pipelines have proven constructive for all teams, students, and collaborators of IMBBC. This operational structure has evolved over the years based on the needs of the facility’s users and the available resources. The practical solutions adopted—from hardware (e.g., depth/breadth balanced structure, user quotas, and temporary storage) to software (e.g., modularized bioinformatics application maintenance and containerization) and human resource management (e.g., frequent intercommunication, continuous cross-discipline training)—reflect IMBBC research to a large extent. However, and by incrementing previous reviews [31], other Institutes and HPC facilities can be informed on the lessons learned (see Section Lessons Learned), and reflect on the computational requirement analysis of the methods presented (see Section Computational Breakdown of the IMBBC HPC-Supported Research) through the spectrum of their own research so as to plan ahead.

HPC facilities could reach a benefit greater than the sum of their capacities once they interconnect. The IMBBC HPC facility lies at the crossroad of 3 RIs, CMBR (Greek node of EMBRC-ERIC), LifeWatchGreece (Greek node of LifeWatch ERIC), and ELIXIR Greece, and via these will pursue further collaboration at larger Tier 0 and Tier 1 levels.

## Data Availability

The data sets supporting the results of this article are available in the following Zenodo repository. The data pertaining to this manuscript are available the repository https://zenodo.org/record/4665308 under a CC BY 4.0 license. The repository is also reachable via the DOI: https://doi.org/10.5281/zenodo.4646132.

## Declarations

### List of abbreviations

ARMS: Autonomous Reef Monitoring Structures; CMBR, Centre for the Study and Sustainable Exploitation of Marine Biological Resources; DE: Differential Expression; eDNA: environmental DNA; GPU: Graphics Processing Unit; HPC: high-performance computing; HTS: high-throughput sequencing; IMBBC: Institute of Marine Biology, Biotechnology and Aquaculture; MPI: Message Passing Interface; SLURM: Simple Linux Utility for Resource Management.

### Competing Interests

The authors declare that they have no competing interests.

### Funding

Funding for the Institute of Marine Biology, Biotechnology and Aquaculture high-performance computing facility was made available through the MARBIGEN project No. FP7-REGPOT-2010-1, the LifeWatchGreece Research Infrastructure (MIS 384676), and the Centre for the Study and Sustainable Exploitation of Marine Biological Resources (CMBR; MIS 5002670) Research Infrastructure, which is implemented under the action “Reinforcement of the Research and Innovation Infrastructure," funded by the Operational Programme Competitiveness, Entrepreneurship, and Innovation (NSRF 2014–2020) and co-financed by Greece and the European Union (European Regional Development Fund).

CMBR has also funded several authors of this study (S.N., A.P., A.A., S.P., J.B.K.). Additional support to H.Z., A.G., and S.N. was provided by the “ELIXIR-GR: Managing and Analysing Life Sciences Data" (MIS: 5002780) project, co-financed by Greece and the European Union's European Regional Development Fund. Additional support to A.A. was provided by Modern Unifying Trends in Marine Biology (MOUNT; MIS 5002470), funded by EPAnEK (“Competitiveness, Entrepreneurship & Innovation" operational programme). E.K. received support from the from the Greater Amberjack project (No. OPF2020). E.K. and E.S. have also been supported from MedAID (Mediterranean Aquaculture Integrated Development, No: 727315, funded by European Union's HORIZON 2020). G.P. and N.P. were supported by LifeWatchGreece, funded by the Greek government under the General Secretariat of Research and Technology, ESFRI (European Strategy Forum on Research Infrastructures) Projects, National Strategic Reference Framework. N.A. received support from the MED UNITS project funded by the European Maritime and Fisheries Fund (EASME/EMFF/2017/1.3.2.2.3/01/); (Study on Advancing fisheries assessment and management advice in the Mediterranean and Black Sea by aligning biological and management units of priority species, funded by the European Maritime and Fisheries Fund); T.D., V.P., and C.S.T. received support from the AQUAEXCEL 2020 infrastructure project (No. 652831, European Union's Horizon 2020); funded by European Union's Horizon 2020; V.P. was also supported by MeagreGEN (“Special Actions, AQUACULTURE", No. 2335, funded by EPAnEK; project (“Special Actions, AQUACULTURE", No. 2335, funded by EPAnEK (“Competitiveness, Entrepreneurship and Innovation, 2014-2020" operational programme)); C.P.'s research was co-financed by Greece and the European Union (European Social Fund- ESF) through the Operational Programme «Human Resources Development, Education and Lifelong Learning» in the context of the project “Reinforcement of Postdoctoral Researchers - 2nd Cycle” (MIS-5033021), implemented by the State Scholarships Foundation (IKY) and she was also supported by the BIOIMAGING-GR, (MIS 5002755, EPANeK), funded by EPAnEK (“Competitiveness, Entrepreneurship and Innova-tion, 2014-2020” operational programme). C.S.T. was also supported by ParaFishControl (No. 634429) and PerformFISH (No. 727610). C.A. received support from LifeWatch European Research Infrastructure Consortium (ERIC).

### Author’s Contributions

E.P., H.Z. and A.G. conceived the study, performed the investigation, curated the data, and administered the project. H.Z. and S.P. worked on visualization. S.N., A.P., J.L., Q.V.H., D.S., P.V., G.P., and N.P. provided software and resources. A.M., C.A., G.K., and C.S.T. were involved in funding acquisition. A.G., H.Z., S.N., A.P., S.P., and E.P. wrote the original draft, and A.G., H.Z., E.P., S.P., N.A., A.A., T.D., E.K., P.K., J.B.K., V.P., C.P., Q.V.H., G.K., T.M., E.S., C.S.T., and C.A. reviewed and edited the original draft. All authors read and approved the final manuscript.

## Supplementary Material

giab053_GIGA-D-21-00111_Original_Submission

giab053_GIGA-D-21-00111_Revision_1

giab053_Response_to_Reviewer_Comments_Original_Submission

giab053_Reviewer_1_Report_Original_Submission Brendan Lawlor -- 5/1/2021 Reviewed

giab053_Reviewer_2_Report_Original_Submission Graciela Dias -- 5/22/2021 Reviewed

giab053_Reviewer_3_Report_Original_Submission Neil Davies, Ph.D. -- 5/22/2021 Reviewed

## References

[1] US Department of Commerce NOaAA. How much water is in the ocean? https://oceanservice.noaa.gov/facts/oceanwater.html. Accessed: 24 November 2020.

[2] Falkowski PG, Fenchel T, Delong EF. The microbial engines that drive Earth’s biogeochemical cycles. Science. 2008; 320(5879):1034–9. 18497287 10.1126/science.1153213

[3] Estes JA, Heithaus M, McCauley DJ, et al. Megafaunal impacts on structure and function of ocean ecosystems. Annu Rev Environ Resour. 2016; 41:83–116.

[4] Arrigo KR. Marine microorganisms and global nutrient cycles. Nature. 2005; 437(7057):349–55. 16163345 10.1038/nature04159

[5] Boero F, Bonsdorff E. A conceptual framework for marine biodiversity and ecosystem functioning. Mar Ecol. 2007; 28:134–45.

[6] Beal LM, De Ruijter WP, Biastoch A, et al. On the role of the Agulhas system in ocean circulation and climate. Nature. 2011; 472(7344):429–36. 21525925 10.1038/nature09983

[7] Remoundou K, Koundouri P, Kontogianni A, et al. Valuation of natural marine ecosystems: an economic perspective. Environ Sci Policy. 2009; 12(7):1040–51.

[8] Bindoff NL, Cheung WWL, Kairo JG, et al. Changing ocean, marine ecosystems, and dependent communities. In: Pörtner H-O, Roberts DC, Masson-Delmotte V, et al. (eds.), IPCC Special Report on the Ocean and Cryosphere in a Changing Climate. IPCC: 2019. In press.

[9] Sala E, Knowlton N. Global marine biodiversity trends. Annu Rev Environ Resour. 2006; 31:93–122.

[10] Tonon T, Eveillard D. Marine systems biology. Front Genet. 2015; 6:181. 26029243 10.3389/fgene.2015.00181 PMC4429633

[11] Dionisi HM, Lozada M, Olivera NL. Bioprospection of marine microorganisms: biotechnological applications and methods. Rev Argent Microbiol. 2012; 44(1):49–60. 22610288 10.1590/S0325-75412012000100010

[12] Tidwell JH, Allan GL. Fish as food: aquaculture’s contribution. EMBO Rep. 2001; 2(11):958–63. 11713181 10.1093/embo-reports/kve236 PMC1084135

[13] Carvalho G, Hauser L. Molecular genetics and the stock concept in fisheries. Rev Fish Biol Fisheries. 1994;4:(3):326–50.

[14] Sakai AK, Allendorf FW, Holt JS, et al. The population biology of invasive species. Annu Rev Ecol Syst. 2001; 32(1):305–32.

[15] Begg GA, Waldman JR. An holistic approach to fish stock identification. Fish Res. 1999; 43(1-3):35–44.

[16] Loreau M. Biodiversity and ecosystem functioning: recent theoretical advances. Oikos. 2000; 91(1):3–17.

[17] Leal MC, Puga J, Serôdio J, et al. Trends in the discovery of new marine natural products from invertebrates over the last two decades–where and what are we bioprospecting?. PLoS One. 2012; 7(1):e30580. 22276216 10.1371/journal.pone.0030580 PMC3262841

[18] Norberg J, Swaney DP, Dushoff J, et al. Phenotypic diversity and ecosystem functioning in changing environments: a theoretical framework. Proc Natl Acad Sci. 2001; 98(20):11376–81. 11535803 10.1073/pnas.171315998 PMC58737

[19] Mardis ER. Next-generation DNA sequencing methods. Annu Rev Genomics Hum Genet. 2008; 9:387–402. 18576944 10.1146/annurev.genom.9.081307.164359

[20] Kulski JK. Next-generation sequencing—an overview of the history, tools, and “omic” applications. In: Kulski JK(ed.), Next generation sequencing: advances, applications and challenges. 2016: 3–60.

[21] Goodwin S, McPherson JD, McCombie WR. Coming of age: ten years of next-generation sequencing technologies. Nat Rev Genet. 2016; 17(6):333. 27184599 10.1038/nrg.2016.49 PMC10373632

[22] Bundy JG, Davey MP, Viant MR. Environmental metabolomics: a critical review and future perspectives. Metabolomics. 2009; 5(1):3–21.

[23] Cahais V, Gayral P, Tsagkogeorga G, et al. Reference-free transcriptome assembly in non-model animals from next-generation sequencing data. Mol Ecol Resour. 2012; 12(5):834–45. 22540679 10.1111/j.1755-0998.2012.03148.x

[24] Baird NA, Etter PD, Atwood TS, et al. Rapid SNP discovery and genetic mapping using sequenced RAD markers. PLoS One. 2008; 3(10):e3376. 18852878 10.1371/journal.pone.0003376 PMC2557064

[25] Tarazona S, García-Alcalde F, Dopazo J, et al. Differential expression in RNA-seq: a matter of depth. Genome Res. 2011; 21(12):2213–23. 21903743 10.1101/gr.124321.111 PMC3227109

[26] Goldford JE, Lu N, Bajić D, et al. Emergent simplicity in microbial community assembly. Science. 2018; 361(6401):469–74. 30072533 10.1126/science.aat1168 PMC6405290

[27] Merelli I, Pérez-Sánchez H, Gesing S, et al. Managing, analysing, and integrating big data in medical bioinformatics: open problems and future perspectives. Biomed Res Int. 2014; 2014. 10.1155/2014/134023 PMC4165507 25254202

[28] Sohn JI, Nam JW. The present and future of de novo whole-genome assembly. Brief Bioinform. 2018; 19(1):23–40. 27742661 10.1093/bib/bbw096

[29] Greene CS, Tan J, Ung M, et al. Big data bioinformatics. J Cell Physiol. 2014; 229(12):1896–900. 24799088 10.1002/jcp.24662 PMC5604462

[30] Pal S, Mondal S, Das G, et al. Big data in biology: the hope and present-day challenges in it. Gene Rep. 2020; 21:100869.

[31] Lampa S, Dahlö M, Olason PI, et al. Lessons learned from implementing a national infrastructure in Sweden for storage and analysis of next-generation sequencing data. Gigascience. 2013; 2(1):2047–17X. 10.1186/2047-217X-2-9 PMC3704847 23800020

[32] Sterling T, Brodowicz M, Anderson M. High performance computing: modern systems and practices. Cambridge, MA 02139, United States: Morgan Kaufmann, 2017.

[33] Wikipedia C. Supercomputing in Europe. https://en.wikipedia.org/w/index.php?title=Supercomputing_in_Europe&oldid=1009652575. Accessed 2 April 2021.

[34] The Members of the PRACE Scientific Steering Committee. The scientific case for computing in Europe 2018–2026. Bristol, UK: Insight Publishers, 2018. https://prace-ri.eu/about/scientific-case/.

[35] Candela L, Castelli D, Pagano P. Virtual research environments: an overview and a research agenda. Data Sci J. 2013; 12:GRDI75–GRDI81.

[36] Haasjes GW. Containerization of legacy applications. https://developer.ibm.com/technologies/containers/articles/containerization-of-legacy-applications/.Accessed: 16 September 2020.

[37] Rad BB, Bhatti HJ, Ahmadi M. An introduction to Docker and analysis of its performance. IJCSNS. 2017; 17(3):228.

[38] Kurtzer GM, Sochat V, Bauer MW. Singularity: scientific containers for mobility of compute. PLoS One. 2017; 12(5):e0177459. 28494014 10.1371/journal.pone.0177459 PMC5426675

[39] Lagnel J, Manousaki T, Kotoulas G, et al. HCMR HPC bioinformatics platform facilitates the marine and aquaculture genomics research in Greece. In: Program and Abstracts of the Hellenic Bioinformatics 09 - 2016 Conference, Hellenic Bioinformatics Society: Thessaloniki, Greece, 2016; 64, abstract no: 26.

[40] Zafeiropoulos H, Gioti A, Ninidakis S, et al. The IMBBC HPC facility: history and configuration, usage statistics, user management and task coordination data and related activities. Zenodo. 2021; doi: 10.5281/zenodo.4665308.

[41] Dongarra JJ, Luszczek P, Petitet A. The LINPACK benchmark: past, present and future. Concurr Comput. 2003; 15(9):803–20.

[42] Castrignanò T, Gioiosa S, Flati T, et al. ELIXIR-IT HPC@CINECA: high performance computing resources for the bioinformatics community. BMC Bioinformatics. 2020; 21(10):352. 32838759 10.1186/s12859-020-03565-8 PMC7446135

[43] Zafeiropoulos H, Viet HQ, Vasileiadou K, et al. PEMA: a flexible pipeline for environmental DNA metabarcoding analysis of the 16S/18S ribosomal RNA, ITS, and COI marker genes. Gigascience. 2020; 9(3):giaa022. doi:10.1093/gigascience/giaa022/5803335. 32161947 10.1093/gigascience/giaa022 PMC7066391

[44] Catchen J, Hohenlohe PA, Bassham S, et al. Stacks: an analysis tool set for population genomics. Mol Ecol. 2013; 22(11):3124–40. 23701397 10.1111/mec.12354 PMC3936987

[45] Varsos C, Patkos T, Oulas A, et al. Optimized R functions for analysis of ecological community data using the R virtual laboratory (RvLab). Biodivers Data J. 2016; 4:e8357. 10.3897/BDJ.4.e8357 PMC5136650 27932907

[46] Katsanevakis S, Coll M, Piroddi C, et al. Invading the Mediterranean Sea: biodiversity patterns shaped by human activities. Front Mar Sci. 2014; 1:32.

[47] Klymus KE, Marshall NT, Stepien CA. Environmental DNA (eDNA) metabarcoding assays to detect invasive invertebrate species in the Great Lakes. PLoS One. 2017; 12(5):e0177643. 28542313 10.1371/journal.pone.0177643 PMC5436814

[48] Bariche M, Al-Mabruk SaA, Ateş MA, et al. New alien Mediterranean biodiversity records (March 2020). Mediterr Mar Sci. 2020; 21(1):129–45.

[49] Obst M, Exter K, Allcock AL, et al. A Marine Biodiversity Observation Network for Genetic Monitoring of Hard-Bottom Communities (ARMS-MBON). Front Mar Sci. 2020; 7:572680.

[50] Pauletto M, Manousaki T, Ferraresso S et al., Genomic analysis of *Sparus aurata* reveals the evolutionary dynamics of sex-biased genes in a sequential hermaphrodite fish. Commun Biol. 2018; 1:119. 10.1038/s42003-018-0122-7 PMC6123679 30271999

[51] Sarropoulou E, Sundaram AYM, Kaitetzidou E, et al. Full genome survey and dynamics of gene expression in the greater amberjack Seriola dumerili. Gigascience. 2017; 6(12): 1–13. 10.1093/gigascience/gix108 PMC5751066 29126158

[52] Tsakogiannis A, Manousaki T, Anagnostopoulou V, et al. The importance of genomics for deciphering the invasion success of the seagrass *Halophila stipulacea* in the changing Mediterranean Sea. Diversity. 2020; 12(7):263.

[53] Danis T, Tsakogiannis A, Kristoffersen JB, et al. Building a high-quality reference genome assembly for the eastern Mediterranean Sea invasive sprinter *Lagocephalus sceleratus* (Tetraodontiformes, Tetraodontidae). bioRxiv. 2020; doi:10.1101/2020.02.17.952580.

[54] Angelova N, Danis T, Jacques L, et al. SnakeCube: containerized and automated next-generation sequencing (NGS) pipelines for genome analyses in HPC environments. Zenodo. 2021; doi: 10.5281/zenodo.4663112.

[55] Natsidis P, Tsakogiannis A, Pavlidis P, et al. Phylogenomics investigation of sparids (Teleostei: Spariformes) using high-quality proteomes highlights the importance of taxon sampling. Commun Biol. 2019; 2:400. 31701028 10.1038/s42003-019-0654-5 PMC6825128

[56] Sarropoulou E, Sepulcre P, Poisa-Beiro L, et al. Profiling of infection specific mRNA transcripts of the European seabass *Dicentrarchus labrax*. BMC Genomics. 2009; 10(1):157. 19361338 10.1186/1471-2164-10-157 PMC2674461

[57] Papadaki M, Kaitetzidou E, Mylonas CC, et al. Non-coding RNA expression patterns of two different teleost gonad maturation stages. Marine Biotechnology. 2020; 22(5):683–95. 32876760 10.1007/s10126-020-09991-2

[58] Warwick RM, Somerfield PJ. All animals are equal, but some animals are more equal than others. J Exp Mar Bio Ecol. 2008; 366(1):184–86.

[59] Arvanitidis CD, Warwick RM, Somerfield PJ, et al. Research Infrastructures offer capacity to address scientific questions never attempted before: are all taxa equal?. PeerJ. 2018. doi:10.7287/peerj.preprints.26819v1.

[60] Vandepitte L, Vanhoorne B, Decock W, et al. A decade of the World Register of Marine Species–general insights and experiences from the Data Management Team: where are we, what have we learned and how can we continue?. PLoS One. 2018; 13(4):e0194599. 29624577 10.1371/journal.pone.0194599 PMC5889062

[61] Gioti A, Siaperas R, Nikolaivits E, et al. Draft genome sequence of a *Cladosporium* species isolated from the Mesophotic Ascidian *Didemnum maculosum*. Microbiol Resour Announc. 2020; 9(18):e00311–20. 32354980 10.1128/MRA.00311-20 PMC7193935

[62] Nikolaivits E, Siaperas R, Agrafiotis A, et al. Functional and transcriptomic investigation of laccase activity in the presence of PCB29 identifies two novel enzymes and the multicopper oxidase repertoire of a marine-derived fungus. Sci Total Environ. 2021; 775:145818. 33631558 10.1016/j.scitotenv.2021.145818

[63] Dagum L, Menon R. OpenMP: an industry standard API for shared-memory programming. IEEE Comput Sci Eng. 1998; 5(1):46–55.

[64] Vouzis PD, Sahinidis NV. GPU-BLAST: using graphics processors to accelerate protein sequence alignment. Bioinformatics. 2011; 27(2):182–8. 21088027 10.1093/bioinformatics/btq644 PMC3018811

[65] Nobile MS, Cazzaniga P, Tangherloni A, et al. Graphics processing units in bioinformatics, computational biology and systems biology. Brief Bioinformatics. 2017; 18(5):870–85. 27402792 10.1093/bib/bbw058 PMC5862309

[66] Mell P, Grance T. The NIST definition of cloud computing. Special Publication (NIST SP) National Institute of Standards and Technology, Gaithersburg, MD: 2011.

[67] Langmead B, Nellore A. Cloud computing for genomic data analysis and collaboration. Nat Rev Genet. 2018; 19(4):208. 29379135 10.1038/nrg.2017.113 PMC6452449

[68] Dahlö M, Scofield DG, Schaal W, et al. Tracking the NGS revolution: managing life science research on shared high-performance computing clusters. GigaScience. 2018; 7:giy028. doi: 10.1093/gigascience/giy028. PMC5928410 29659792

