# Supplementary material for: 0s and 1s in marine molecular research: a regional HPC perspective
Source: Gigascience. 2021 Aug 18;10(8):giab053. doi: 10.1093/gigascience/giab053 (PMC8371273; doi:10.1093/gigascience/giab053)

GigaScience

0s and 1s in marine molecular research: a regional HPC perspective

--Manuscript Draft--

**Manuscript Number:**	GIGA-D-21-00111R1
**Full Title:**	0s and 1s in marine molecular research: a regional HPC perspective
**Article Type:**	Review
**Funding Information:**	FP7 Research Potential of Convergence Regions (FP7-REGPOT-2010-1) — Dr Antonios Magoulas<br>General Secretariat for Research and Technology (GR) (MIS 384676) — Dr Christos Arvanitidis<br>European Regional Development Fund () (MIS 5002670) — Dr Antonios Magoulas
**Abstract:**	High-performance computing (HPC) systems have become indispensable for modern marine research, providing support to an increasing number and diversity of users. Pairing with the impetus offered by high-throughput methods to key areas such as non-model organism studies, their operation continuously evolves to meet the corresponding computational challenges.<br>Here we present a Tier-2 (regional) HPC facility, operating for over a decade at the Institute of Marine Biology, Biotechnology, and Aquaculture (IMBBC) of the Hellenic Centre for Marine Research in Greece. Strategic choices made in design and upgrades aimed to strike a balance between depth (the need for a few high-memory nodes) and breadth (a number of slimmer nodes), as dictated by the idiosyncrasy of the supported research. An in-depth computational requirement analysis of the latter revealed the diversity of marine fields, methods and approaches adopted to translate data into knowledge. In addition, hardware and software architectures, usage statistics, policy and user management aspects are presented.<br>Drawing upon the last decade's experience from the different levels of operation of the IMBBC HPC facility, a number of lessons are presented; these have contributed to the facility's future directions, in the light of emerging distribution technologies (e.g. containers) and Research Infrastructure evolution. In combination with detailed knowledge of the facility usage and its upcoming upgrade, future collaborations in marine research and beyond are envisioned.
**Corresponding Author:**	Evangelos Pafilis<br>Hellenic Center for Marine Research<br>Heraklion Crete, GREECE
**Corresponding Author Secondary Information:**	
**Corresponding Author's Institution:**	Hellenic Center for Marine Research
**Corresponding Author's Secondary Institution:**	
**First Author:**	Haris Zafeiropoulos
**First Author Secondary Information:**	
**Order of Authors:**	Haris Zafeiropoulos<br>Anastasia Gioti<br>Stelios Ninidakis<br>Antonis Potirakis<br>Savvas Paragkamian<br>Nelina Angelova<br>Aglaia Antoniou

Powered by Editorial Manager® and ProduXion Manager® from Aries Systems Corporation

	Theodoros Danis
	Elisavet Kaitetzidou
	Panagiotis Kasapidis
	Jon Bent Kristoffersen
	Vasileios Papadogiannis
	Christina Pavloudi
	Quốc Việt Hà
	Jacques Lagnel
	Nikos Pattakos
	Giorgos Perantinos
	Dimitris Sidirokastritis
	Panagiotis Vavilis
	Georgios Kotoulas
	Tereza Manousaki
	Elena Sarropoulou
	Costas Tsigenopoulos
	Christos Arvanitidis
	Antonios Magoulas
	Evangelos Pafilis
**Order of Authors Secondary Information:**	
**Response to Reviewers:**	We would like to kindly thank the reviewers for the time they spent to thoroughly read our manuscript. We appreciate all their accurate comments, which helped at the improvement of our manuscript. In the revised version, we have addressed all of the reviewer's comments and suggestions and have incorporated changes/amendments to the manuscript (indicated in cyan), where necessary. Below we cite our detailed answers to the editor and reviewers' comments and suggestions (reviewer text starts with a hash #).<br><br># Reviewer reports:<br># The paper is a retrospective on 12 years of running what has become a regional HPC facility in Greece. It reviews # the gradual evolution of a single-server resource into a Tier-2 facility, gives some insights into how the facility is<br># organized and run, highlights some of the research done using the facility, and offers some lessons learned from # these 12 years. In this, it should be of interest to researchers who use HPC facilities and those who run them.<br># The paper is well written and well organized, making it very accessible even to non-specialists. There are a<br># number of observations that I would make with a view to improving the relevance of the paper, and a minor<br># correction for readability.<br><br># Reviewer #1 comment #1:<br># 1. Containerization: This is perhaps one of the more important recent developments in the HPC space (and<br># beyond). It is treated in a number of sections where, if I understand correctly, Singularity is the adopted<br># platform. #But what is the standard image format used? Singularity for direct compatibility with the HPC<br># environment, or #Docker for eventual external compatibility (e.g. with cloud compute)? What reasoning led to<br># any decisions here.

	We chose Singularity as the standard image format for Zorba, while In terms of locally-developed containers, we support both technologies. Singularity was preferred because it ensures compatibility with our job scheduling system (i.e. SLURM). Singularity has been developed to perform complex applications on HPC environments focusing specifically on the needs of the scientific community and aiming to support reproducible science in a simple way. Therefore, it has a completely different approach compared to Docker regarding the namespace (allowing to setup a container without privileged operations, so that a non-root user can act as root inside a container without being root on the host outside) and filesystems, and supports Message Passing Interface (MPI). Docker, on the other hand, is not designed for data processing, but for running microservices (e.g. web-services). Moreover, Singularity is compatible with Docker containers, allowing to take advantage of all the benefits of Docker; e.g the large number of already developed containers and its larger community. Thus, Singularity is commonly used by Academic and Research institutions as the containerization technology for their HPC-oriented containers [1].<br>We have clarified that we use Singularity in the corresponding section (lines 157-158). While Singularity is our container technology of choice regarding running containers in our HPC facility, we also employ Docker for external compatibility purposes, i.e. the containers we create and share. The newly added last paragraph of Section "5D. The way forward: Develop locally, share and deploy centrally" and section "6. Cloud Computing" elaborate on such Docker use and its synergy with Cloud/HPC systems.<br><br>Reference<br>[1] Kurtzer, Gregory M., Vanessa Sochat, and Michael W. Bauer. "Singularity: Scientific containers for mobility of compute." PloS one 12.5 (2017): e0177459.<br><br><br># It would be very interesting to hear the authors' opinion on what developments they expect, or hope for, with<br># regards to containerization.<br><br>Containers are an already established technology, some of the largest cloud providers worldwide (e.g. Google Cloud, Azure, Amazon Web Services) have already adopted them to a great extent. Container use is also on the rise in research; it is our belief that it will be further accelerated in the future, especially in the context of FAIRification efforts. Despite "indirect costs" related to these efforts, such as costs to containerize legacy software, we believe that these technologies will become the norm. Ongoing, community-driven efforts, such as the BioContainers project (https://biocontainers.pro/) that provides both the infrastructure and the basic guidelines to build, manage and distribute images and containers, play a great part towards this direction. Additional contributions in the same direction are provided by a number of groups, institutes and universities that have already started to containerize and distribute already-developed and commonly known bioinformatics software tools (e.g the Hurwitz Lab container repository (https://github.com/hurwitzlab?q=singularity+OR+docker)). We have added a comment on our view regarding the future of containers at the end of section "5D. The way forward: Develop locally, share and deploy centrally" (lines 530 - 545).<br><br><br># Reviewer #1 comment #2:<br># 2. Cloud computing: Commercial cloud providers and the potential for hybridization with traditional "in-house"<br># HPC, is another topic that is relevant today for research that requires significant computation. This was touched<br># on very briefly in the first of the Lessons Learned sub-sections, where budget and containerization were<br># mentioned. I suspect that other HPC managers and staff would welcome some more detail here. What do the<br># authors consider to be the factors that would lead towards the (increased) use of cloud computing? What are<br># the drawbacks? Had any comparison of costs been undertaken? Are there any mismatches between the HPC<br># model of containerization, and that of commercial cloud providers? (e.g. Docker vs Singularity) that would need # to be addressed?

Powered by Editorial Manager® and ProduXion Manager® from Aries Systems Corporation

There is a trend for cloud computing services managed by a web interface. This is mainly because they offer simplicity and high availability to users with less or even no experience in HPC systems. In addition, the time needed for data manipulation, software installation and user-system interaction is significantly reduced compared to a local HPC facility. On the other hand, tool experimentation and benchmarking is more limited in cloud computing compared to local facilities, which are in general more dedicated to specific research areas. While a cost comparison e.g. among the reported HPC usage and equivalent solutions in the cloud is possible, a thorough one has not been conducted. Since our facility still relies on legacy (i.e. not containerized) software and workflows, we are in a preparatory phase towards an era where cloud resources can be optimally used, and thus an exhaustive cost comparison is not relevant at this stage for Zorba. The more progress is made, the more such a strategic shift would make sense in the future. In addition, non-profit, academia-oriented, cloud computing solutions will be explored first prior to commercial ones. Regarding mismatches between the two systems on containerization: Although both fully support Singularity containers, Singularity has been specifically developed to support HPC systems, while Docker is designed to run in isolated environments (i.e. Virtual machines), as explained in comment 1. As elaborated further in comment 4 and by the means of comprehensiveness, a paragraph on “6. Cloud computing” has been added in the manuscript.

Reviewer #1 comment #3:
3. Multi-threaded programming: The authors correctly mention the use of software threads as the general
solution to parallelization in bioinformatics programs. But there is a problem in the way that they characterize
multi-node parallelization. The authors point out that to distribute parallel tasks over multiple nodes, threading
is not enough. The typical solution for bioinformatic programmers is to use MPI (across nodes) alongside
OpenMP (within nodes). The authors point out that this is only used in a small minority of bioinformatic
applications, and that MPI usage is low in others. As a reviewer, I presume that this is an indication, from the
authors, that cross-node parallelization (i.e. fully distributed) is not something that happens often on their
facility, or in any case is considered to be niche enough to influence the choice of node types on the cluster
(presumably in favour of nodes with large numbers of cores). Firstly, could I ask the authors if I have correctly
interpreted the message of the subsection entitled "Software optimizations for parallel execution"?

The reviewer has correctly understood that cross-node parallelization does not happen often on our facility, mainly because there is a limited number of tools that exploit similarly well cores from multiple nodes (multi-node parallelization). This has indeed partially influenced our strategic choices on the system architecture.

If so, I would like to offer another perspective on the matter, and would welcome the authors' thoughts. OpenMP
and MPI programming is difficult. They are both low-level abstractions that force the coder to concentrate on
implementation details close to the OS and hardware. I believe it is *this* difficulty that leads to this approach
being used only in niche settings, and that if easier approaches to multi-node distribution were available,
bioinformaticians would make use of them. Such approaches do exist (actor-based distribution as exemplified by
the Akka library is one) but they are not generally known to bioinformaticians. They offer high-level abstractions

Powered by Editorial Manager® and ProduXion Manager® from Aries Systems Corporation

that hide the details of both intra-node threading and inter-node Inter Process Communication. If
bioinformaticians were introduced to these techniques, it is possible that they could make more efficient use of
HPC (and/or cloud) infrastructure.

We agree that MPI and (in some cases when numerous dependencies exist) OpenMP require more in-depth knowledge and experience in how to capitalize on multi-core systems (mostly MPI, since OpenMP is less complex). Actor-based parallel model is indeed a more understandable way of producing concurrency and could become popular among bioinformaticians. We thank the reviewer for the suggestion; it could be further explored once a pertinent IMBBC HPC use case arises.

I think this is a chicken-and-egg situation, where users of HPC facilities are encouraged to use MPI/OpenMP,
and HPC facilities operate as if these low-level protocols were the only available approach. The authors mention
"training as an integral component to the HPC mindset". Do they see an opportunity here to use that training to
extend the computational reach of bioinformaticians?

The vast majority of Zorba users make use of third-party software tools (not developing their own) for their workflows, thus the Zorba team's primary care so far has been to render users more familiar with how different parallel technologies work, so as to understand how to use these more efficiently in terms of hardware resources. Once efficient and user-friendly models for parallelization become widely acceptable in the community, we do foresee adopting them and training Zorba users in these. In addition, The Zorba team and many researchers in the Institute actively follow related training activities (e.g. by ELIXIR, EOSC), which are commonly based on user surveys to identify current needs and future goals in terms of computing.

#Reviewer #1 comment #4:
#4. Network intensive processing: The first Lessons Learned subsection explores the extent to which processes are
memory, or CPU bound. No mention is made of network intensity. In a way this point extends from the previous
one about fully-distributed processes, and it also touches on the cloud vs HPC question. It might be said that the
distinguishing feature of a HPC facility (over say a private or public cloud) is the InfiniBand network layer. So the
question of making full use of this feature is relevant. I'd welcome some discussion on the general point of HPC
# vs cloud in the paper, and about InfiniBand's role in that comparison, especially in the section that looks to the
future of the facility.

A large number of bioinformatics tools and housekeeping software running on Zorba are network-intensive, either due to I/O operations they perform in shared filesystems, or to node intercommunication. To address this issue we adopted IB, as mentioned in the companion preprint paper (https://zenodo.org/record/4665308, see section "A2. The Zorba configuration of the IMBBC HPC facility" therein). The reviewer is right that we did not mention network intensity in the main text. Since it is indeed important for the discussion, we have added relevant information in the revised manuscript, lines 132 - 137.
Infiniband (IB) is the key element to efficiently serve intercommunication among structural parts of an HPC facility (either traditional or cloud-based); therefore, investment in high bandwidth (based on RDMA technology) is important. In practise, processes using MPI are those that really take advantage of IB, mainly in traditional HPC systems when running MPI among a large number of nodes. In cloud infrastructures, IB is also important in cases when resources from different nodes are combined to provide a unified virtual machine. However, most commercial Cloud

Powered by Editorial Manager® and ProduXion Manager® from Aries Systems Corporation

providers do not support IB interconnection. In that case, fast direct memory access between the nodes is needed in order to ensure a similar or equal result in terms of performance, as if it ran on a single server. However, IB cannot really accelerate IO operations (e.g. file transfers, data reads and writes) since it is bounded by the filesystem type, the disks and the concurrent demand at a specific moment. Some of the above points are mentioned in our revised manuscript, new paragraph “6. Cloud computing”.

#Reviewer #1 comment #5:
5. Minor point - the paper has references to numbered sections (e.g. "Section 2") but there is no numbering in
the paper format.

Section numbering has been updated.

#Reviewer #2 comment #1:
The manuscript presents a description and importance of High-performance computing (HPC) in marine
research. The topic is of interest because allows important studies in marine research. The manuscript describes
the system architecture and capacity of processing of Zorba facility. I think that the important point of the Zorba
facility is concern a accessible communication between users and administrators. The method is quite standard
and is appropriate for the study, especially given that the main focus of the paper is to describe the evolution of
IMBBC HPC facility during the 12 past years. Is notable the progress of the facility in the analysis of a wide
variety of information. The conclusions are satisfactory. The solutions applied in hardware (depth/breadth
balanced structure, user quotas and temporary storage), software (modularized bioinformatics application
maintenance and containerization are very interesting) and training enable that the scientific community can
deal with a wide # variety of information. The language is clear and does not require a heavy edition and the
statistical methods are not relevant for the present paper.

We would like to thank the reviewer for the positive comments.

#Reviewer #2 comment #2:
Minor revisions
In the topic "Computational breakdown of the IMBBC HPC supported research", please fix the order of the
figures. It seems to me that is Figure 4 and not Figure 3. In the phrases "As shown in Fig. 3...", "Long
computational times (Fig 3A)", "... approaches is the significant storage limitations (Fig 3C)..." Figure 4 is not
clearly labeled, please see the text of 4B and 4C.

All mismatched annotations have been fixed in the revised manuscript.

Reviewer #3 comment #1:
The manuscript provides an accessible and informative review of the evolving needs and solutions for High
Performance Computing at a regional scale serving users in the broad domain of marine science and
biotechnology. For readers without deep expertise in computational science (as is the case for this reviewer) the
authors are commended in providing a useful insight to the challenges facing such

Powered by Editorial Manager® and ProduXion Manager® from Aries Systems Corporation

	cyberinfrastructure and how<br># those challenges have been addressed in the past as well as some future guidance. The use cases provided were<br># appropriate and illustrative of the types of study supported by this HPC facility, and the heterogenous demands<br># for types of computing resource and how these can be met and balanced among the needs of different users.<br># The common challenge of storage was well expounded but solutions appear to be still rather elusive.<br><br>We would like to thank the reviewer for the positive comments. We agree that storage is a major challenge and is not easy to be met. A storage upgrade scheduled within 2021 (see Section "7. Future Directions") is expected to alleviate current storage challenges in Zorba. However, given the ever increasing data production (e.g. as the result of decreasing sequencing costs, and/or of rising imaging technologies), responsible storage use approaches as described here remain only partial solutions to anticipated future storage needs. Centralized (Tier-1 or higher) storage solutions represent a longer-term solution, which is in line with current views on how to handle big data generated by international research consortia in a long-lasting manner.<br>We have added the above comments in the revised manuscript, in section 5B "Quota... overloaded" lines 465 - 475.
**Additional Information:**	
**Question**	**Response**
Are you submitting this manuscript to a special series or article collection?	No
**Experimental design and statistics**<br><br>Full details of the experimental design and statistical methods used should be given in the Methods section, as detailed in our Minimum Standards Reporting Checklist. Information essential to interpreting the data presented should be made available in the figure legends.<br><br>Have you included all the information requested in your manuscript?	Yes
**Resources**<br><br>A description of all resources used, including antibodies, cell lines, animals and software tools, with enough information to allow them to be uniquely identified, should be included in the Methods section. Authors are strongly encouraged to cite Research Resource Identifiers (RRIDs) for antibodies, model organisms and tools, where possible.	Yes

Powered by Editorial Manager® and ProduXion Manager® from Aries Systems Corporation

Have you included the information requested as detailed in our Minimum Standards Reporting Checklist?	
**Availability of data and materials**<br><br>All datasets and code on which the conclusions of the paper rely must be either included in your submission or deposited in publicly available repositories (where available and ethically appropriate), referencing such data using a unique identifier in the references and in the “Availability of Data and Materials” section of your manuscript.<br><br>Have you have met the above requirement as detailed in our Minimum Standards Reporting Checklist?	Yes

Powered by Editorial Manager® and ProduXion Manager® from Aries Systems Corporation

Manuscript

Click here to access/download;Manuscript;main_first_revision.pdf

GigaScience, 2017, 1–12

doi: xx.xxxx/xxxx
Manuscript in Preparation
REVIEW

REVIEW

0s and 1s in marine molecular research: a regional HPC perspective

Haris Zafeiropoulos[1,2,*], Anastasia Gioti[1,*], Stelios Ninidakis[1], Antonis Potirakis[1], Savvas Paragkamian[1,2], Nelina Angelova[1], Aglaia Antoniou[1], Theodoros Danis[1,3], Eliza Kaitetzidou[1], Panagiotis Kasapidis[1], Jon Bent Kristoffersen[1], Vasileios Papadogiannis[1], Christina Pavloudi[1], Quoc Viet Ha[4], Jacques Lagnel[5], Nikos Pattakos[1], Giorgos Perantinos[1], Dimitris Sidirokastritis[6], Panagiotis Vavilis[6], Georgios Kotoulas[1], Tereza Manousaki[1], Elena Sarropoulou[1], Costas S Tsigenopoulos[1], Christos Arvanitidis[1,7], Antonios Magoulas[1] and Evangelos Pafilis[1,†]

[1]Hellenic Centre for Marine Research (HCMR), Institute of Marine Biology, Biotechnology and Aquaculture (IMBBC), Former U.S. Base of Gournes, P.O. Box 2214, 71003, Heraklion, Crete, Greece and [2]Department of Biology, University of Crete, Voutes University Campus, P.O.Box 2208, 70013, Heraklion, Crete, Greece and [3]Greece School of Medicine, University of Crete and [4]BULL SAS, rue du gros caillou, 78340 Les Clayes-sous-Bois, France and [5]INRAE, UR1052, Génétique et Amélioration des Fruits et Légumes (GAFL), 67 Allée des Chênes, Centre de Recherche PACA, Domaine Saint Maurice, CS60094, 84143 Montfavet, France and [6]Hellenic Centre for Marine Research (HCMR), Network Operation Center, Former U.S. Base of Gournes, P.O. Box 2214, 71003, Heraklion, Crete, Greece and [7]LifeWatch ERIC, Sector II-III Plaza de España, 41071, Seville, Spain

[*]Contributed equally
[†]Corresponding author: pafilis@hcmr.gr

Abstract

Background High-performance computing (HPC) systems have become indispensable for modern marine research, providing support to an increasing number and diversity of users. Pairing with the impetus offered by high-throughput methods to key areas such as non-model organism studies, their operation continuously evolves to meet the corresponding computational challenges.
Results Here we present a Tier-2 (regional) HPC facility, operating for over a decade at the Institute of Marine Biology, Biotechnology, and Aquaculture (IMBBC) of the Hellenic Centre for Marine Research in Greece. Strategic choices made in design and upgrades aimed to strike a balance between depth (the need for a few high-memory nodes) and breadth (a number of slimmer nodes), as dictated by the idiosyncrasy of the supported research. Qualitative computational requirement analysis of the latter revealed the diversity of marine fields, methods and approaches adopted to translate data into knowledge. In addition, hardware and software architectures, usage statistics, policy and user management aspects of the facility are presented.
Conclusions Drawing upon the last decade's experience from the different levels of operation of the IMBBC HPC facility, a number of lessons are presented; these have contributed to the facility's future directions, in the light of emerging distribution technologies (e.g. containers) and Research Infrastructure evolution. In combination with detailed knowledge of the facility usage and its upcoming upgrade, future collaborations in marine research and beyond are envisioned.

Key words: marine research; high performance computing (HPC); containerization; computational requirements; high-throughput sequencing (HTS); research infrastructures (RIs); biodiversity; biotechnology; aquaculture

1. Background

The ubiquitous marine environments (more than 70% of the global surface [1]) mold Earth's conditions to a great extent. The interconnected abiotic [2] and biotic factors (from Bacteria [2] to megafauna [3]), shape biogeochemical cycles [4] and climate [5, 6] from a local to the global scale. In addition, marine systems have high socio-economic value [7] as an essential source of food and by supporting renewable energy and transport among other services [8]. The study of marine environments involves a series of disciplines (scientific fields); from Biodiversity [9] and Oceanography to (eco)systems biology [10], and from Biotechnology [11] to Aquaculture [12].

To shed light on the evolutionary history of (commercially important) marine species [13] as well as on how invasive species respond and adapt to novel environments [14] the analysis of their genetic stock structure is fundamental [15]. Similarly, biodiversity assessment is essential to elucidate ecosystem functioning [16] and to identify taxa with potential for bioprospecting applications [17]. Furthermore, systems biology approaches provide both a theoretical and a technical background for integrative analyses to flourish [18]. However, conventional methods do not offer the information needed to explore the aforementioned scientific topics.

High - throughput sequencing (HTS) and sister methods have launched a new era in many biological disciplines [19, 20]. These technologies allowed access to the genetic, transcript, protein and metabolite repertoire [21] of studied taxa or populations, and facilitated the analysis of organism-environment interactions in communities and ecosystems [22]. Whole Genome Sequencing (WGS) and Whole Transcriptome Sequencing (WTS) approaches provide valuable information for the study of non-model taxa [23]. This information can be further enriched by genotyping-by-sequencing approaches, for instance Restriction site-associated DNA sequencing (RAD-seq) [24], or by investigating gene expression dynamics through Differential Expression (DE) analyses [25]. Moving from single species to assemblages, molecular-based identification and functional profiling of communities has become available through marker, genome or transcriptome sequencing from environmental DNA (eDNA) (metabarcoding, metagenomics, metatranscriptomics) [26]. These methods address the problem of *"how to produce and get access to the information?"* on different biological systems and molecules to a great extent.

These 0's and 1's of information (i.e the data) come along with challenges regarding their management, analysis and integration [27]. The computational requirements for these tasks by far exceed the capacity of a standard laptop/desktop by far, owing to the sheer volume of the data and to the computational complexity of the bioinformatic algorithms employed for their analysis. For example, building the *de novo* genome assembly of a non-model Eukaryote may require algorithms of nondeterministic polynomial time ((NP)-complete problem) complexity. This analysis can reach up to several hundreds or thousands of GB of memory (RAM) [28]. Hence, the challenges of *"how to exploit all these data?"* and *"how to transform data into knowledge"* set the present framework in biological research [29, 30].

To address these computational challenges, the use of High-Performance Computing (HPC) systems has become essential in life sciences and systems biology [31]. HPC is the scientific field that aims at the optimal incorporation of technology, methodology, and application thereof to achieve *"the greatest computing capability possible at any point in time and technology"* [32]. Such systems range from a small number to several thousands of interconnected computers (compute nodes). According to the Partnership foR Advanced Computing in Europe (PRACE), the European HPC facilities are categorized in: a. European Centres (Tier-0), b. national (Tier-1) and c. regional (Tier-2) centres [33]. As PRACE highlights, *"computing drives science and science drives computing"* in a great range of scientific fields; from the endeavor to maintain a sustainable Earth, to the efforts for expanding the frontiers in our understanding of the universe [34]. On top of the heavy computational requirements, biological analyses come with a series of other practical issues that often affect the bioinformatics-oriented HPC systems.

Researchers with purely biological background often lack the coding skills or even the familiarity required for working with Command Line Interfaces (CLI) [34]. Virtual Research Environments (VREs) are web - based e - services platforms, particularly useful for researchers lacking expertise or / and computing resources [35]. Another common issue is that most analyses include a great number of steps, with the software used in each of these having equally numerous dependencies. Lack of continuous support for tools with different dependencies, as well as frequent and non-periodical versioning of the latter, often results in broken links and further compromises the reproducibility of analyses [36]. Widely-used containerization technologies, e.g. Docker [37] and Singularity [38] ensure reproducibility of software and replication of the analysis, thus partially addressing these challenges. By encapsulating software code along with all its corresponding dependencies in such containers, software packages become reproducible in any operating system in an easy-to-download-and-install fashion, on any infrastructure.

The Institute of Marine Biology Biotechnology and Aquaculture (IMBBC) has been developing a computing hub which, in conjunction with national and European Research Infrastructures (RIs), to support state of the art marine research. The regional IMBBC HPC facility allows processing of data that derive from the Institute's sequencing platforms and expeditions, and from multiple external sources in the context of interdisciplinary studies. Here, we present insights from a thorough analysis of the research supported by the facility and some of its latest usage statistics in terms of resource requirements, computational methods and data types; the above have contributed in shaping the facility along its lifespan.

2. The IMBBC HPC facility
from a single server to a Tier-2 system

The IMBBC HPC facility was launched in 2009 to support the computational needs over a range of scientific fields in marine biology, with a focus on non-model taxa [39]. The facility was initiated as an infrastructure of the hitherto Institute of Marine Biology and Genetics (IMBG) of the Hellenic Centre for Marine Research (HCMR). Its development has followed the development of national RIs (Fig 1, also see [40] Section A1). The first nodes were used to support the analysis of datasets generated from methods such as eDNA metabarcoding and multiple omics. Since 2015, the facility also supports VREs, including e-services and virtual laboratories (vLabs). The current configuration of the facility presented herein is named *Zorba* (box 4 in Fig. 1) and will be upgraded within 2021 (see Section 7). Hereafter, *Zorba* refers to the specific system setup from 2015 and onwards, while the facility throughout its lifespan will be referred to as "IMBBC HPC".

Compiled on: July 6, 2021.
Draft manuscript prepared by the author.

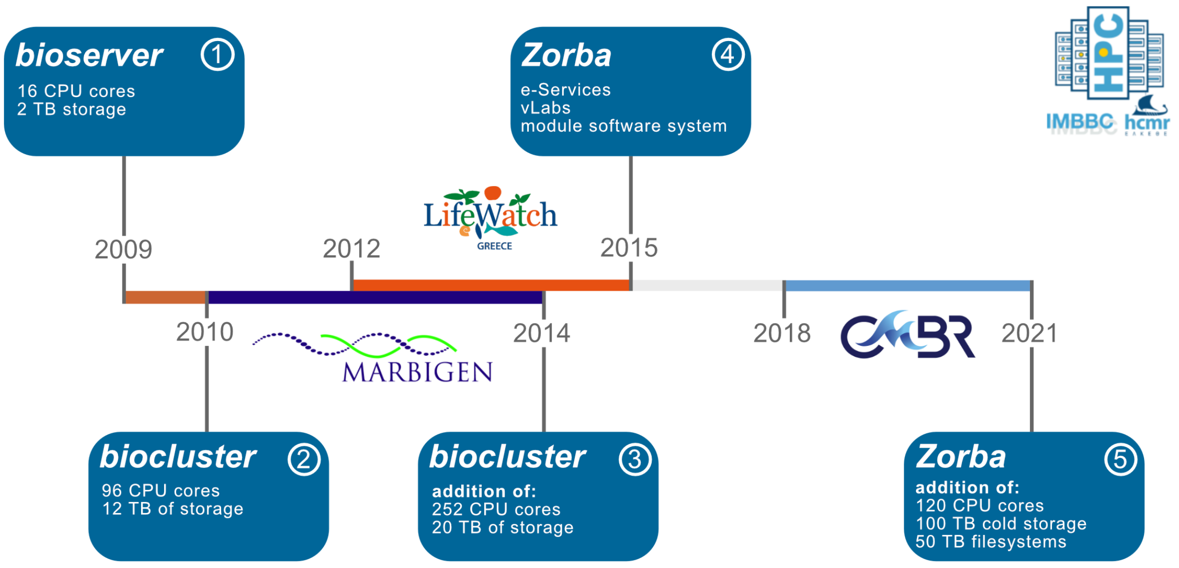

Figure 1. Evolution of the IMBBC HPC facility during the past 12 years, with hardware upgrade (blue boxes) and funding milestones (logos of RIs) highlighted. A single server that launched the bioinformatics era in 2009 evolved to the current Tier-2 system *Zorba* (box 4), which allows processing a wide variety of information from DNA sequences to biodiversity data. Different names of the facility denote distinct system architectures.

Zorba currently consists of 328 CPU cores, 2.3 TB total memory and 105 TB storage. Job submission takes place on the four available computing partitions, or queues, as explained in Fig. 2. *Zorba* at its current state achieves a peak performance of 8.3 trillion double-precision floating-point operations per second, or 8.3 Tflops, as estimated by LinPack benchmarking [41]. On top of these, a total 7.5 TB is distributed to all servers for the storage of environment and system files. Interconnection of both the compute and login nodes takes place via an infiniband (IB) interface of 40 Gbps capacity, which features very high throughput and very low latency. Infiniband is also used for a switched interconnection between the servers and the four available file systems. A thorough technical description of *Zorba* is available at (see [40] Section A2).

More than 200 software packages are currently installed and available to users at *Zorba*, covering the most common analysis types. These tools allow assembly, HTS data preprocessing, phylogenetic tree construction, ortholog finding, population structure modeling, to name a few. Access to these packages is provided through Environment Modules, a broadly-used means of accessing software in HPC systems [42].

During the last two years, *Zorba* has been moving from system - dependent pipelines previously developed at IMBBC (e.g. ParaMetabarCoding) towards containerization of available and new pipelines/tools. A complete metabarcoding analysis tool for various marker genes (PEMA) [43], the chained and automated use of STACKS, software for population genetics analysis from short-length sequences [44] (latest version), a set of statistical functions in R for the computation of biodiversity indices and analyses in cases of high computational demands [45], as well as a programming workflow for the automation of biodiversity historical data curation (DECO) are among the in-house developed containers. The standard container / image format used on *Zorba* is Singularity. Singularity images can be served by any *Zorba* partition; Docker images can run instantly as Singularity images. A thorough description of the software containers developed in *Zorba* can be found in the [40] Section D.

Zorba daily function is ensured by a core team of four full-time experienced staff: a hardware officer, two system administrators, and a permanent researcher in biodiversity informatics and data science.

More than 70 users (internal and external scientists), investigators, postdoctoral researchers, technicians, and doctoral / postgraduate students have gained access to the HPC infrastructure until today. Support is provided officially through a helpdesk ticketing system. An average of 31 requests/month have been received (since June 2019), with the most demanded categories being troubleshooting (38.2%) and software installation (23.8%). Since October 2017, monthly meetings among HPC users have been established to regularly discuss such issues.

Proper scheduling of the submitted jobs and fair resource sharing is a major task that needs to be confronted day-to-day. To address this, a specific usage policy, for each of the various partitions, and a scheduling software tool set have been adopted in *Zorba*. Policy terms are dynamically adapted to the HPC hardware architecture and to the usage statistics, with revisions being discussed between the HPC core team and users. Simple Linux Utility for Resource Management (SLURM) open-source cluster management system orchestrates the job scheduling along with the allocation of resources and a booking system helps users to organize their projects and administrators to monitor the resource reservations on a mid-long term basis. A SLURM Database Daemon (slurmdbd) has also been installed to allow logging and recording of job usage statistics into a separate SQL database (see PREPRINT). An extended description of user and job administration and orchestration can be found at (see [40] Section C1).

Training is an integral component of the HPC facility mindset since its launch and enables knowledge sharing across MSc, PhD students and researchers within and outside the Institute. Introductory courses are organized on a regular basis, aiming at familiarizing new users with Unix environments, programming, and HPC usage policy and resource allocation (e.g. job submission in SLURM). Furthermore, the IMBBC HPC facility has served, since 2011, as an international training platform for specific types of bioinformatic analyses (see [40] Section C2). For instance, the facility has provided computational resources for workshops on Microbial diversity, Genomics and

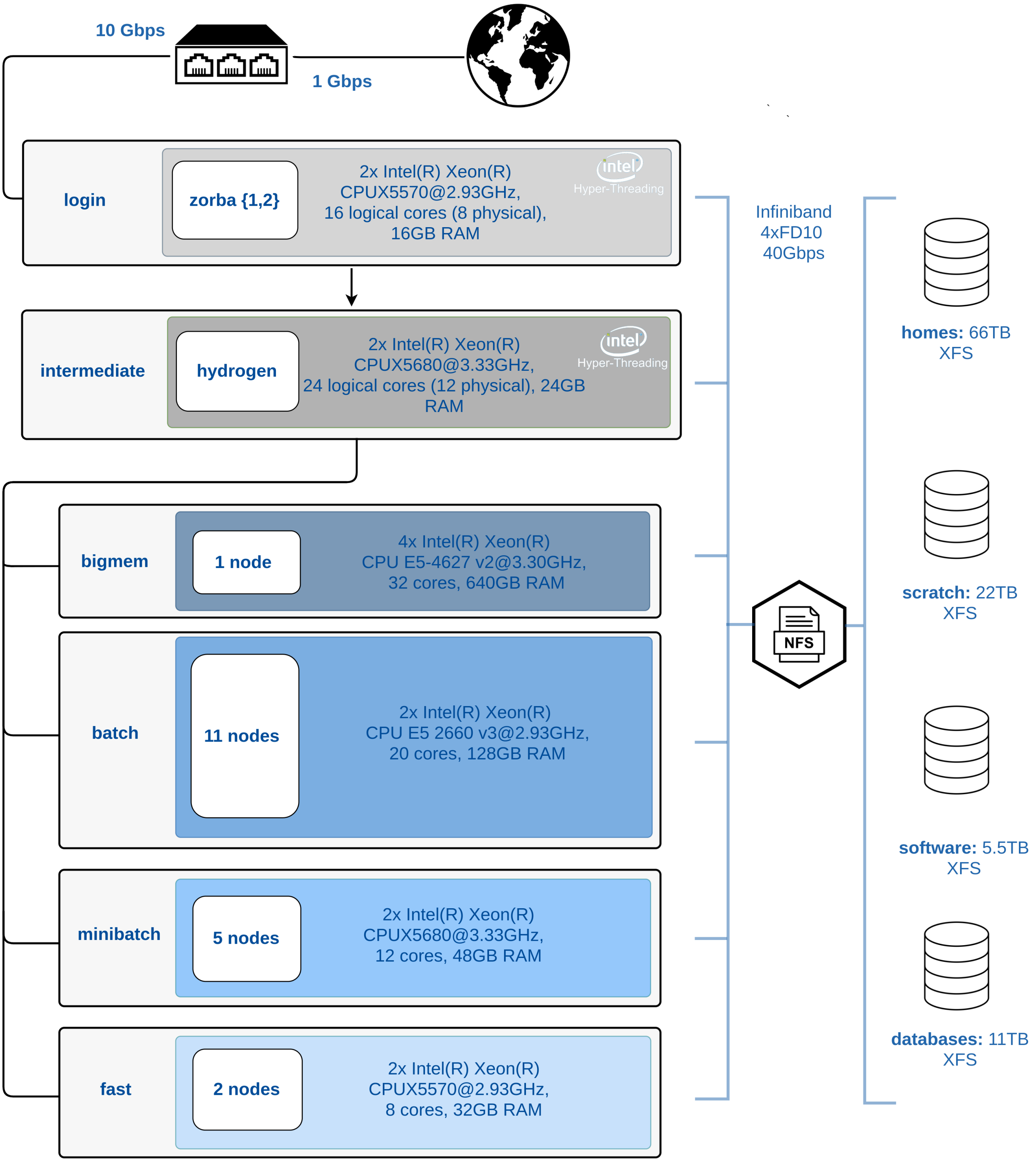

Figure 2. Block diagram of the *Zorba* architecture. This is the IMBBC HPC facility architecture in its current setup, after 12 years of development. There are 2 login nodes and one intermediate where users may develop their analyses. Computational nodes are split into 4 partitions with different specs and policy terms: `bigmem` supporting processes requiring up to 640 GB RAM, `batch` handling mostly (but not exclusively) parallel-driven jobs (either in a single node or across several nodes), `minibatch` aiming to serve parallel jobs with reduced resource requirements and fast partition for non-intensive jobs.All servers, except file systems, run Debian 9 (kernel 4.9.0-8-amd64).

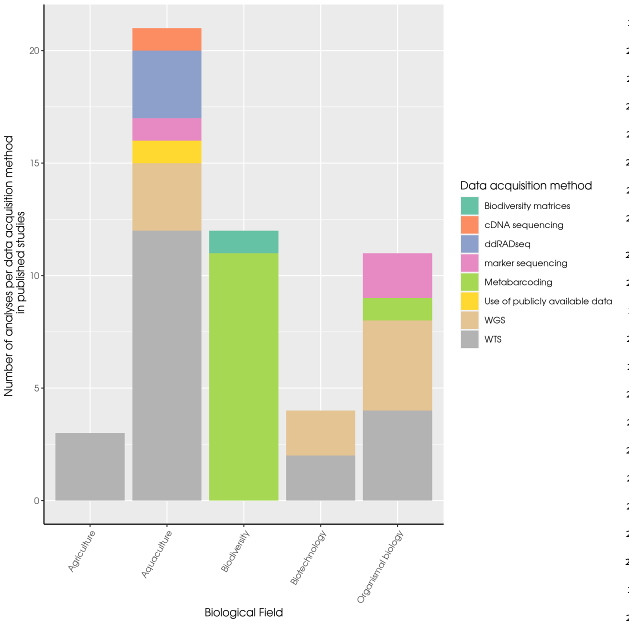

Figure 3. Bar chart with the number of publications that have used IMBBC HPC facility resources, grouped per scientific field. The different methods for data acquisition are also presented. WGS: whole-genome sequencing; WTS: whole-transcriptome sequencing.

Metagenomics, Genomics in Biodiversity, Next Generation Sequencing technologies and informatics tools for studying marine biodiversity and adaptation in the long term, or Ecological Data Analysis using R (ECODAR). The plan is to enhance and diversify the educational component of the HPC facility by providing courses on a more permanent basis and targeting a larger audience. An extensive listing of training activities is given at [40] Section C2.

3. Computational breakdown of the IMBBC HPC-supported research

Systematic labelling of IMBBC HPC-supported published studies (n=47) was performed to highlight their resource requirements. Each study was manually labelled with the relevant scientific field, the data acquisition method, the computational methods, and its resource requirements; all the annotations were validated by the corresponding authors (see [40] Section D2). It should be stated that the conclusions of this overview are specific to the studies conducted at IMBBC.

The scientific fields of Aquaculture (~40% of studies), Biodiversity (~26% of studies) and Organismal biology (~19% of studies) account for the majority of the research publications supported by the IMBBC HPC facility (Fig. 3 and [40] Supplementary file 1).

On the other hand, studies in the Biotechnology and Agriculture fields indicate contemporary and beyond-marine orientations of research at IMBBC, respectively (see [40] Section B2). In addition, eight methods of data acquisition (experimental or in silico) have been defined (Fig. 3). Among these methods, WGS and WTS have been widely used in multiple fields (Biotechnology, Organismal Biology, Aquaculture). Conversely, ddRAD sequencing has been solely employed for population genetic studies in the context of Aquaculture.

The 47 published studies employed different computational methods (sets of tasks executed on the HPC facility). These studies served different purposes, from a range of bioinformatics analyses to HPC-oriented software optimization. The computational methods were categorized in eight classes (Fig. 4). The resource requirements of each computational method were evaluated in terms of memory usage, computational time and storage. Reflecting the current *Zorba* capacity, studies which, in any part of their analysis, exceeded 128 GB of memory or/and 48 hours of running time or/and 200 GB physical space were classified as studies with high demands (see [40] Supplementary file: `imbbc_hpc_labelling_data.xlsx`).

As shown in Fig. 4, the two most commonly used computational methods have rather different resource requirements. While "differential expression (DE) analysis" shows a notable trend for both long computational time (Fig. 4A), and high memory (Fig. 4B), "eDNA-based community analysis" does not have high resource requirements either in computation time or memory. High memory was commonly associated with computational methods including de novo assembly; all relevant research concerned non-model taxa and involved short-read sequencing or combinations of short- and long-read sequencing. By contrast, "phylogenetic analysis" did not involve intensive RAM use; this is largely due to the fact that software used by IMBBC users adopts parallel solutions for tree construction. Long computational times (Fig. 4A) were most often observed at the functional annotation step in "transcriptome analysis", "DE analysis" and "comparative and evolutionary omics", when this step involved BLAST queries of thousands of predicted genes against large databases such as nr (NCBI). Finally, a common challenge emerging from all bioinformatic approaches is the significant storage limitations (Fig. 4C); this challenge was associated with the use of HTS technologies that produce large raw data, and the analysis of which involves creation of numerous intermediate files.

Overall, published studies using the IMBBC HPC facility show a degree of variance with respect to the types of tools used (depending on the user, its bioinformatic literacy, and other factors), each of which is more or less optimized with respect to HPC use. Moreover, the variance in computational needs observed within each type of computational method, reflects the diversity of the studied taxonomic groups. For instance, "transcriptome analysis" (involving *de novo* assembly and functional annotation steps) was employed for the study of taxa as diverse as Bacteria, sponges, Fungi, fish and goose barnacles. The complexity of each of these organisms' transcriptomes can explain to a large extent the differences observed in computational time, memory and storage.

Furthermore, *Zorba* CPU and RAM statistics collected since 2019 allowed observing some overall patterns: An average computation load per month of less than or close to 50% of its max capacity (50% of 236 kilocorehours/month) for most (20) out of the 24 months of the logging period was one of these. Memory requirements were also heterogeneous: most (90%) of a 44K jobs performed in the same 24 month period, required less than 10 GB of RAM. 0.30% of the jobs required more than 128 GB of RAM, i.e. exceeding the memory capacity of the main compute nodes (`batch` partition). The detailed usage statistics of *Zorba* are described in [40] Section B1 and Supplementary file: `zorba_usage_statistics.xlsx`.

4. Scientific impact stories

Below, some examples of research results that were made possible with the IMBBC HPC facility are described. This list of "use cases" is by no means exhaustive, but rather an attempt to highlight different fields of research supported by the facility along with their distinct computational features.

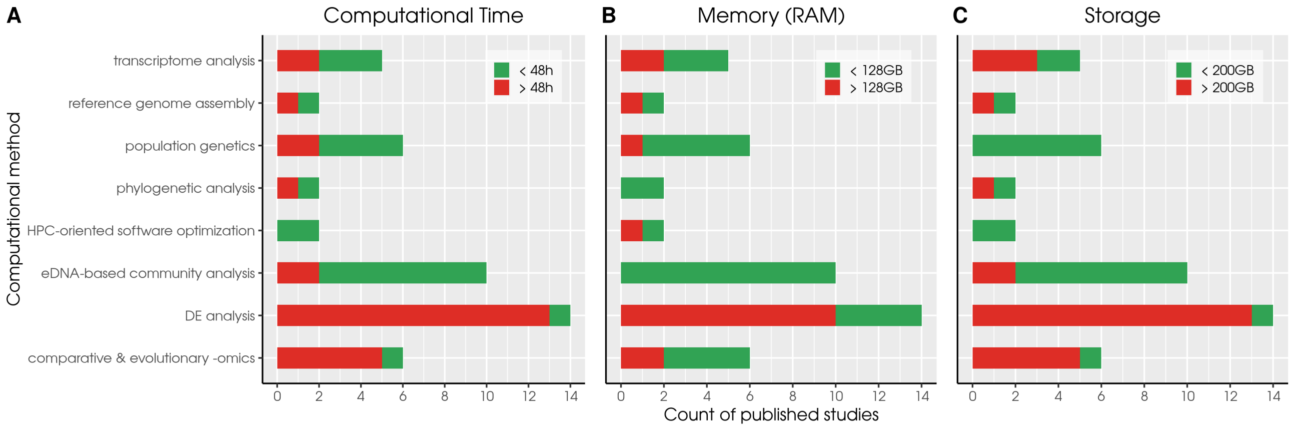

Figure 4. Resource requirements of the various computational methods employed at the IMBBC HPC facility to support published research A) long computational time (>48h), B) high memory (>128 G B)high storage requirements (>200 GB) and C) red color denotes studies with high requirement for a certain HPC feature. For instance, all eDNA-based community analyses performed at *Zorba* until now have not required long computational time.

A. Invasive species range expansion detected with eDNA data from ARMS

The Mediterranean biodiversity and ecosystems are experiencing profound transformations owing to Lessepsian migration, international shipping, and aquaculture, which lead to the migration of nearly 1000 alien species [46]. The first step towards addressing the effects of these invasions is the monitoring of the introduced taxa. eDNA metabarcoding has proved a powerful tool in this direction, allowing detection of invasive species [47], often preceding macroscopic detection. One such example is the first record of the nudibranch *Anteaeolidiella lurana* (Ev. Marcus & Er. Marcus, 1967) in Greek waters in 2020 [48]. eDNA metabarcoding analysis allowed detecting the species with high confidence on fouling communities developed on Autonomous Reef Monitoring Structures (ARMS). This finding, confirmed with image analysis of photographic records on a later deployment period, is an example of work conducted within the framework of the European ASSEMBLE plus programme (ARMS-MBON). PEMA software [43] was used in this study as well as in the 30-month pilot phase of ARMS-MBON [49].

B. Providing omics resources for large genome-size, non-model taxa

Zorba has been used for building and annotating numerous de novo genome and transcriptome assemblies of marine species such as the gilthead sea bream *Sparus aurata* [50] or the greater amberjack *Seriola dumerili* [51]. Both genome and transcriptome assemblies of species with large genomes often exceed the maximum available memory limit, eventually affecting the strategic choices for *Zorba* future upgrades (see Section 7). For instance, building the draft genome assembly of the seagrass *Halophila stipulacea* (estimated genome size 3.5 GB) using Illumina short reads has been challenging even for seemingly simple tasks such as a kmer analysis [52]. Taking advantage of short and long-read sequencing technologies to construct high-quality reference genomes, the near-chromosome level genome assembly of *Lagocephalus sceleratus* (Gmelin, 1789) was recently completed, as a case study of high ecological interest due to the species' successful invasion throughout the Eastern Mediterranean [53]. In the context of this study, an automated containerized pipeline allowing high-quality genome assemblies from Oxford Nanopore and Illumina data was developed (SnakeCube, [54]). The availability of standardized pipelines offers great perspectives for in-depth studies of numerous marine species of interest in aquaculture and conservation biology, including rigorous phylogenomic analyses to position each species in the tree of life (e.g.[55]).

C. DE analysis of aquaculture fish species sheds light on critical phenotypes

Distinct observable properties such as morphology, development, and behavior, characterize living taxa. The corresponding phenotypes may be controlled by the interplay between specific genotypes and the environment. To capture an individual's genotype at a specific time point, molecular tools for transcript quantification have followed the fast development of technologies, with Expressed Sequence Tags (EST) being the first approach to be historically used, especially suited for non-model taxa [56]. Nowadays, the physiological state of aquaculture species is retrieved through investigation of stage-specific and immune- and stress response-specific transcriptomic profiles using RNAseq. The corresponding computational workflows involve installing various tools at *Zorba* and implementing a series of steps that often take days to compute. These analyses, besides detecting transcripts at a specific physiological state, have successfully identified regulatory elements such as microRNAs. Through the construction of a regulatory network with putative target genes, microRNAs have been linked to the transcriptome expression patterns. The most recent example is the identification of microRNAs and their putative target genes involved in ovary maturation [57].

D. Large-scale ecological statistics: Are all taxa equal?

The nomenclature of living organisms, as well as their description and their classification under a specific nomenclature code, have been studied for more than two centuries. Up to now, all the species present in an ecosystem are considered *equal*, in terms of their contribution to diversity. However, this "axiome" has been tested only once before, on the UK's marine animal phyla, showing the inconsistency of the traditional Linnaean classification between different major groups [58]). In [59] the average taxonomic distinctness index ($\Delta+$) and its variation (*Lambda+*) were calculated on a matrix deriving from the complete World Register of Marine Species (WoRMS) [60], containing more than 250,000 described species of marine animals. It is the R-vLab web application along with its HPC high RAM back-end components (on `bigmem`, see Section 2) that

made such a calculation possible. This is the first time such a hypothesis is tested on a global scale. Preliminary results show that the two biodiversity indices exhibit complementary patterns and that there is a highly significant, yet non-linear relationship between the number of species within a phylum and the average distance through the taxonomic hierarchy.

E. Discovery of novel enzymes for bioremediation

Polychlorinated biphenyls (PCBs) are complex, recalcitrant pollutants that pose a serious threat to wildlife and human health. Identification of novel enzymes that can degrade such organic pollutants is intensively studied in the emerging field of bioremediation. In the context of the Horizon 2020 TASCMAR project, global ocean sampling provided a large biobank of fungal invertebrate symbionts, and through large-scale screening and bioreactor culturing, a marine-derived fungus able to remove a PCB compound was identified for the first time. *Zorba* resources and domain expertise in fungal genomics were used as a CMBR service for the analysis of multi-omic data for this symbiont. Following genome assembly of *Cladosporium sp.* TM-S3 [61], transcriptome assembly and phylogenetic analysis revealed the full diversity of the symbiont's multicopper oxidases, enzymes commonly involved in oxidative degradation [62]. Among these, two laccase-like proteins shown to remove up to 71% of the PCB compound are now being expressed to optimize their use as novel biocatalysts. This step would not have been possible without the annotation of the *Cladosporium* genome with transcriptome data; mapping of the purified enzymes' LC-MS spectra against the set of predicted proteins allowed to identify their corresponding sequences.

5. Lessons learned

A. Depth and breadth are both required for a bioinformatics - oriented HPC

In our experience, the vast majority of the analyses run at the IMBBC HPC infrastructure are CPU-intensive. RAM-intensive jobs (>128 GB RAM, see Section 3) represent only ~0.3% of the total jobs executed over the last 2 years (see [40] Section B1). Despite the difference in frequency of executed jobs with distinct requirements, serving both types of jobs and ensuring their successful completion is equally important for addressing fundamental marine research questions (as shown in Section 3). The need for both HPC depth (the need for a few high-memory nodes) and breadth (a number of slimmer nodes) has been previously reported [31]. This need reflects the idiosyncrasy of different bioinformatics analysis steps, often even within the same workflow. High-memory nodes are cannot-do-without for tasks such as *de novo* assembly of large genomes, while the availability of as many as possible less powerful nodes can speed up the execution of less demanding tasks, and free resources for other users to compute. Future research directions and the available budget further dictate tailoring of the HPC depth and breadth. Cloud-based services, e.g. for containerized workflows, may also facilitate this process once these become more affordable.

B. Quota... overloaded

We observed that independently of the type of analysis, storage was an issue for all *Zorba* users (Fig. 4). A high percentage of these issues relate to the raw data from HTS projects. These data are permanently stored in the home directories, occupying significant space. This, in conjunction with the fact that users delete their data with great reluctance, makes storage one of the major issues of daily use in *Zorba*. In specific cases where users' quota was exceeded uncontrollably, the *Zorba* team has been applying compression of raw and output data in contact with the user, but this is by no means a stable strategy. More generally, the performance of the existing storage configuration in *Zorba* being close to reaching its limits with the increase in users and its concurrent use, several solutions have been adopted to resolve the issue. The most long-lasting solution has been the adoption of a per - user quota system to allow storage sustainability and fairness in our allocation policy. This quota system constitutes nevertheless a limiting factor in pipeline execution, since lots of software tools produce unpredictably too many intermediate files, which not only increase storage but also cause job failures due to space restrictions. We managed the above issue by adding a `scratch` filesystem as an intermediate storage area for the runtime capacity needs. Following completion of their analysis, users retain only the useful files and the rest are permanently removed. A storage upgrade scheduled within 2021 (see Section 7) is expected to alleviate current storage challenges in Zorba. However, given the ever increasing data production (e.g. as the result of decreasing sequencing costs, and/or of rising imaging technologies), responsible storage use approaches as described here remain only partial solutions to anticipated future storage needs. Centralized (Tier-1 or higher) storage solutions represent a longer-term solution, which is in line with current views on how to handle big data generated by international research consortia in a long-lasting manner.

C. Continuous intercommunication among different disciplines matters

Smooth function of an HPC system and exploitation of its full potential for research requires stable employment of a core team of computer scientists and engineers, in close collaboration with an extended team of researchers. At least four disciplines are involved in *Zorba*-related issues: Computer scientists, Engineers, Biologists (in the broad sense, including ecologists, genomicists, etc.), and Bioinformaticians with varying degrees of literacy in Biology and Informatics and various domain specializations (comparative genomics, biodiversity informatics, bacterial metagenomics, etc). Continuous communication among representatives of these four disciplines has been substantial to research supported by *Zorba* and to the evolution of the HPC system itself over time. In our experience, an HPC system cannot function effectively and for long without full-time system administrators, nor with bioinformaticians alone. Although it has not been the case since the system's onset, investment in monthly meetings, seminars, and training events (in biology, containers, domain-specific applications, and computer science, see Section 2) are the only way to establish stable intercommunication among different players of an HPC system. Such proximity translates into timely and adequate systems and bioinformatics analysis support; an element that in its turn translates into successful research (See Section 3). It should be noted that the overall good experience in connectivity among different HPC "players" derives from *Zorba* being a Tier-2 system, with a number of active permanent users in the order of tens. The establishment of such inter-communication was relatively straightforward to implement with periodic meetings and the assistance of ticketing and other management solutions (see [40] Section C1).

D. The way forward: Develop locally, share and deploy centrally

The various approaches regarding the function of an HPC system are strongly related to the different viewpoints of the academic communities towards the relatively new disciplines of Bioinformatics and Big Data. These approaches are strongly affected by national and international decisions that affect the ability to fund supercomputer systems. There are advantages in deploying bioinformatics - oriented HPC systems in centralized (Tier-0, Tier-1) facilities. Better prices at hardware purchases, easier access to HPC-tailored facilities, for instance in terms of the cooling system and physical space, or experienced technical personnel (see also [31]). However, synergies between regional (Tier-2) and centralized HPC systems are fundamental for moving forward in supporting the diverse and demanding needs of bioinformatics. An example of such synergies concerns technical solutions (e.g. containerization) that address long-standing software sharing issues. In our experience, a workflow/pipeline can be developed by experts within the context of a specific project in a regional HPC facility. Once a production version of the pipeline is packaged, it can be distributed to centralized systems to cover a broader user audience (see Section 2). Singularity containers have been developed to utterly suit HPC environments, mostly because they permit root access of the system in all cases. In addition, Singularity is compatible with all Docker images and can be used with Graphics Processing Units (GPUs) and Message Passing Interface (MPI) applications. This is why we chose to run containers in a Singularity format at *Zorba*. However, as Docker containers are widely used, especially in cloud computing (see more about cloud computing in Section 6), workflows and services produced at IMBBC are offered in both container formats. Containers are an already established technology, used by the biggest cloud providers worldwide and increasingly by non-profit research Institutes. Despite “indirect costs” (e.g costs to containerize legacy software) we believe that these technologies will become the norm in the future, especially in the context of reproducibility and interoperability of bioinformatics analysis.

E. Software optimizations for parallel execution

The most common ways of achieving implicit or explicit parallelization in modern multicore systems for Bioinformatics, Computational Biology, and Systems Biology software tools, are the software threads - provided by programming languages - and/or the OpenMP API [63]. These types of multiprocessing make good use of the available cores on a multicore system (single node), but they are not capable of combining the available CPU cores from more than one node. Some other software tools use MPI to spawn processing chunks to many servers and/or cores, or (even better) combine MPI with OpenMP/Threads to maximize the parallelization in Hybrid models of concurrency. Such designs are now used to a great extent in some cases, such as phylogeny inference software that makes use of Monte Carlo Markov Chain (MCMC) samplers. However, these cases are but a small number compared to the majority of bioinformatics tasks, while their usage in other analyses is low. At the hardware level, simultaneous multithreading is not enabled in the compute nodes of the IMBBC HPC infrastructure. Since the majority of analyses running on the cluster demand dedicated cores, hardware multithreading does not perform well. In our experience, the existence of more (logical) cores in compute nodes misleads the least experienced users into using more threads than the physically available ones, which slows down their executions. On the other hand, assisting servers (filesystems, login nodes, web servers) make use of hardware multithreading, since they serve numerous small tasks from different users/sources which commonly contain I/O operations. GPUs provide an alternative way for parallel execution, but they are supported by a limited number of bioinformatics software tools. Nevertheless, GPUs can optimize the execution process in specific, widely-used bioinformatic analyses, such as sequence alignment [64, 65], image processing in microtomography (e.g. microCT), or basecalling of Nanopore raw data.

6. Cloud computing

A recent alternative to traditional HPC systems, such as the one described in this review, is cloud computing. Cloud computing is the way of organizing computing resources so they are provided over the Internet ("cloud"). This paradigm of computing requires the minimum management effort possible [66]. Cloud computing providers exist in both commercial vendors and academic/publicly-funded institutions and infrastructures (for more on cloud computing for bioinformatics, [67]). Computing resources can be reserved from individuals, institutions, organizations or even scientific communities. The most widely-known commercial cloud providers are the "big three" of cloud computing, namely Amazon Web Services (AWS), Google Cloud Platform and Microsoft Azure, while other cloud vendors are constantly emerging. Academic/publicly-funded providers are also available, e.g. the EMBL-EBI Embassy Cloud.

Cloud computing services are increasingly adopted in research, mainly because they offer simplicity and high availability to users with reduced or even no experience in HPC systems, through web interfaces. For this type of users, time needed for data manipulation, software installation and user-system interaction is significantly reduced compared to using a local HPC facility.

Container technologies, especially Docker, along with container - management systems such as Kubernetes combined with OpenStack have been widely used in a number cloud computing systems, in particular in the research domain.It should be noted, however, tool experimentation and benchmarking is more limited in cloud computing compared to local facilities, and is costly, since it demands additional corehours of segmented computation. In-house HPC infrastructures can be fully configured to suit specific research area needs (storage available, fast interconnection for MPI jobs, number of CPUs versus available RAM, assisting services, etc.). Moreover, in cases where InfiniBand (IB) interconnection, a computer networking communications standard, is adopted in HPC, the performance in jobs and software that take advantage of it is substantial. Given the features and advantages of each approach (mentioned above) one could foresee the scenario of combining them to address the research community needs.

7. Future Directions

An upgrade of the existing hardware design of *Zorba* has been scheduled in 2021, funded by the CMBR RI (Fig. 1). More specifically:

i. 3 nodes of 40 CPU physical cores will be added through new partitions (120 cores in total)
ii. the total RAM will be increased by 3.5 TB
iii. 100 TB of cold storage will be installed and is expected to alleviate the archiving problem at the existing homes/scratch file systems
iv. the total usable existing storage capacity for users in home and scratch partitions will be increased by approxi-

mately 100 TB

With this upgrade, it is expected that the total computational power of *Zorba* will be increased by approximately 6 TFlops, while the infrastructure will be capable of serving memory-intensive jobs requiring up to 1.5 TB of RAM, hosted on a single node. Eventually, more users will be able to concurrently load and analyze big datasets on the filesystems. Over the coming 2 years, *Zorba* is also expected to have two major additions:

i. the acquisition of a number of GPU nodes to build a new partition especially for serving software that has been ported to run on GPUs
ii. the design of a parallel file system (Ceph or Lustre) to optimize concurrent I/O operations to speed up CPU-intensive jobs.

The expectation is that the upcoming upgrade of *Zorba* will further enhance collaborations with external users, since the types of bioinformatic tasks supported by the infrastructure are common to other disciplines beyond marine science, such as environmental omics research in the broad term. A nationalwide survey targeting the community of researchers studying the environment and adopting the same approaches (HTS, biodiversity monitoring) has revealed that their computational and training needs are on the rise (A. Gioti et al., unpublished observations). Usage peaks and valleys were observed in *Zorba* (see[40] Section B1), similarly to other HTS-oriented HPC systems [31]. It is therefore feasible to share *Zorba* idling time with other scientific communities. Besides, the *Zorba* upgrade comes very timely in a period where additional computational infrastructures emerge: The Cloud infrastructure EG-CI, funded by the Greek node of ELIXIR, is currently at the preproduction phase. It will constitute a national Tier-1 HPC facility, designed to host ~50 computational nodes of different capabilities (regular servers, GPU-enabled servers, SSD-enabled servers, etc), and provide users the option to either create custom Virtual Machines for their computational services or to upload and execute workflows of containerised scientific software packages. In this context, a strategic combination of *Zorba* and EG-CI capabilities is expected to build a strong computational basis in Greece. It is also expected that *Zorba* functionality will be augmented through its connection with the Super Computing Installations of LifeWatch ERIC (e.g. Picasso facility in Malaga, Spain). Building upon the lessons learned of the last twelve years, a foreseeable challenge for the facility is the enhancement of its usage monitoring to the example of international HPC systems [68], in order to allow even more efficient use of computational resources.

8. Conclusions

Zorba is an established Tier-2 HPC regional facility operating in Crete, Greece. It serves as an interdisciplinary computing hub in the eastern Mediterranean, where studies in marine conservation, invasive species, extreme environments, and aquaculture are of great scientific and socio-economic interest. The facility has supported, since its launch over a decade ago, a number of different fields of marine research, covering all kingdoms of life; it can also share part of its resources to support research beyond the marine sciences.

The operational structure of *Zorba* enables continuous communication between users and administrators for more effective user support, troubleshooting and job scheduling. More specifically, training, regular meetings and containerization of in-house pipelines have proven constructive for all teams, students and collaborators of IMBBC. This operational structure has evolved over the years based on the needs of the facility's users and the available resources. The practical solutions adopted, from hardware (e.g. depth/breadth balanced structure, user quotas and temporary storage), to software (e.g. modularized bioinformatics application maintenance and containerization) and human resource management (e.g. frequent intercommunication, continuous cross-discipline training) reflect IMBBC research to a large extent. However, and by incrementing previous reviews [31], other Institutes and HPC facilities can be informed on the lessons learned (see Section 5), and reflect on the computational requirement analysis of the methods presented (see Section 3) through the spectrum of their own research so as to plan ahead.

HPC facilities could reach a benefit greater than the sum of their capacities once they interconnect. The IMBBC HPC facility lies at the crossroad of three RIs, CMBR (Greek node of EMBRC-ERIC), LifeWatchGreece (Greek node of LifeWatch ERIC) and ELIXIR Greece, and will pursue via these further collaboration at larger Tier-0 and Tier-1 levels.

Availability of supporting data and materials

The data sets supporting the results of this article are available in the following Zenodo repository.

Declarations

List of abbreviations

ARMS: Autonomous Reef Monitoring Structures ; CLI: Command Line Interfaces ; DE: Differential Expression ; eDNA: environmental DNA ; EST: Expressed Sequence Tags ; GPUs: Graphics Processing Units ; HCMR: Hellenic Centre for Marine Research ; HPC: high performance computing ; HTS: high-throughput sequencing ; IMBBC: Institute of Marine Biology, Biotechnology and Aquaculture ; IMBG: Institute of Marine Biology and Genetics ; MCMC: Monte Carlo Markov Chain ; MPI: Message Passing Interface ; NP: nondeterministic polynomial ; PCBs: Polychlorinated biphenyls ; RAD-seq: Restriction site-associated DNA sequencing ; RIs: research infrastructures ; SLURM: Simple Linux Utility for Resource Management ; vLabs: virtual laboratories ; VREs: Virtual Research Environments ; WGS: Whole Genome Sequencing ; WTS: Whole Transcriptome Sequencing ; WoRMS: World Register of Marine Species

Ethical Approval (optional)

Not applicable.

Consent for publication

Not applicable.

Competing Interests

The authors declare that they have no competing interests.

Funding

Funding the IMBBC HPC facility was made available through the MARBIGEN project No. FP7-REGPOT-2010-1, the LifeWatchGreece Research Infrastructure (MIS 384676) and the Centre for the study and sustainable exploitation of Marine

Biological Resources (CMBR) (MIS 5002670) Research Infrastructure, which is implemented under the Action "Reinforcement of the Research and Innovation Infrastructure", funded by the Operational Programme Competitiveness, Entrepreneurship and Innovation (NSRF 2014-2020) and co-financed by Greece and the European Union (European Regional Development Fund).

CMBR has also funded several authors of this study (SN, AP, AA, SP, JK). Additional support to HZ, AG, SN was provided by the "ELIXIR-GR: Managing and Analysing Life Sciences Data" (MIS: 5002780) project, co-financed by Greece and the European Union - European Regional Development Fund. Additional support to AA was provided by the MOdern UNifying Trends in marine biology (MOUNT) (MIS 5002470) funded by EPAnEK. EK received support from the from the Greater Amberjack project (No. OPF2020). GP, NP were supported by the LifeWatchGreece, funded by the Greek Government under the General Secretariat of Research and Technology (GSRT), ESFRI Projects, National Strategic Reference Framework (NSRF). NA received support from the MED UNITS project; TD, VP, CT from the AQUAEXCEL 2020 research infrastructure project (No. 652831); VP was also supported by MeagreGEN; CP was supported by the BIOIMAGING-GR research infrastructure (MIS 5002755). CST was also supported by ParaFishControl (No. 634429) and PerformFISH (No. 727610). CA received support from LifeWatch ERIC.

Author's Contributions

E.P., H.Z. and A.G. conceived the study, performed investigation, data curation, and project administration. H.Z. and S.P. worked on visualization. S.N., A.P., J.L., QV.H, D.S., P.V., G.P., and N.P. provided software and resources. A.M., C.A., G.K., and CS.T. were involved in funding acquisition. A.G., H.Z., S.N., A.P., S.P. and E.P. wrote the original draft, and A.G., H.Z., E.P., S.P., N.A., A.A., T.D., E.K., P.K., JB.K., V.P., C.P., QV.H, G.K., T.M., E.S., CS.T., and C.A. provided reviews and editing to the original draft. All authors read and approved the final manuscript.

Acknowledgements

The authors would like to thank Dr. Pantelis Topalis IMBB/FORTH for fruitful discussions on HPC strategic design. We would also like to thank the ELIXIR-GR Compute platform and the Resource Allocation Committee for advice and ideas exchange.

We would also like to thank Dr. Ioulia Santi, Dr. Anastasios Bounas and Romanos Siaperas for their feedback on the systematic labelling of the published studies used for this review.

References

1. US Department of Commerce NOaAA, How much water is in the ocean?; 2021. https://oceanservice.noaa.gov/facts/oceanwater.html.
2. Falkowski PG, Fenchel T, Delong EF. The microbial engines that drive Earth's biogeochemical cycles. science 2008;320(5879):1034–1039.
3. Estes JA, Heithaus M, McCauley DJ, Rasher DB, Worm B. Megafaunal impacts on structure and function of ocean ecosystems. Annual Review of Environment and Resources 2016;41:83–116.
4. Arrigo KR. Marine microorganisms and global nutrient cycles. Nature 2005;437(7057):349–355.
5. Boero F, Bonsdorff E. A conceptual framework for marine biodiversity and ecosystem functioning. Marine Ecology 2007;28:134–145.
6. Beal LM, De Ruijter WP, Biastoch A, Zahn R. On the role of the Agulhas system in ocean circulation and climate. Nature 2011;472(7344):429–436.
7. Remoundou K, Koundouri P, Kontogianni A, Nunes PA, Skourtos M. Valuation of natural marine ecosystems: an economic perspective. environmental science & policy 2009;12(7):1040–1051.
8. Bindoff NL, Cheung WWL, Kairo JG, Arístegui J, Guinder VA, Hallberg R, et al. Changing Ocean, Marine Ecosystems, and Dependent Communities. In: IPCC Special Report on the Ocean and Cryosphere in a Changing Climate; 2019.https://www.ipcc.ch/site/assets/uploads/sites/3/2019/11/09_SROCC_Ch05_FINAL.pdf.
9. Sala E, Knowlton N. Global marine biodiversity trends. Annu Rev Environ Resour 2006;31:93–122.
10. Tonon T, Eveillard D. Marine systems biology. Frontiers in genetics 2015;6:181.
11. Dionisi HM, Lozada M, Olivera NL. Bioprospection of marine microorganisms: biotechnological applications and methods. Revista Argentina De Microbiologia 2012 Mar;44(1):49–60.
12. Tidwell JH, Allan GL. Fish as food: aquaculture's contribution. EMBO reports 2001;2(11):958–963.
13. Carvalho G, Hauser L. Molecular genetics and the stock concept in fisheries. In: Molecular genetics in fisheries Springer; 1995.p. 55–79.
14. Sakai AK, Allendorf FW, Holt JS, Lodge DM, Molofsky J, With KA, et al. The population biology of invasive species. Annual review of ecology and systematics 2001;32(1):305–332.
15. Begg GA, Waldman JR. An holistic approach to fish stock identification. Fisheries research 1999;43(1-3):35–44.
16. Loreau M. Biodiversity and ecosystem functioning: recent theoretical advances. Oikos 2000;91(1):3–17.
17. Leal MC, Puga J, Serôdio J, Gomes NC, Calado R. Trends in the discovery of new marine natural products from invertebrates over the last two decades–where and what are we bioprospecting? PLoS One 2012;7(1):e30580.
18. Norberg J, Swaney DP, Dushoff J, Lin J, Casagrandi R, Levin SA. Phenotypic diversity and ecosystem functioning in changing environments: a theoretical framework. Proceedings of the National Academy of Sciences 2001;98(20):11376–11381.
19. Mardis ER. Next-generation DNA sequencing methods. Annu Rev Genomics Hum Genet 2008;9:387–402.
20. Kulski JK. Next-generation sequencing—an overview of the history, tools, and "omic" applications. Next generation sequencing-advances, applications and challenges 2016;p. 3–60.
21. Goodwin S, McPherson JD, McCombie WR. Coming of age: ten years of next-generation sequencing technologies. Nature Reviews Genetics 2016;17(6):333.
22. Bundy JG, Davey MP, Viant MR. Environmental metabolomics: a critical review and future perspectives. Metabolomics 2009;5(1):3–21.
23. Cahais V, Gayral P, Tsagkogeorga G, Melo-Ferreira J, Ballenghien M, Weinert L, et al. Reference-free transcriptome assembly in non-model animals from next-generation sequencing data. Molecular ecology resources 2012;12(5):834–845.
24. Baird NA, Etter PD, Atwood TS, Currey MC, Shiver AL, Lewis ZA, et al. Rapid SNP discovery and genetic mapping using sequenced RAD markers. PloS one 2008;3(10):e3376.
25. Tarazona S, García-Alcalde F, Dopazo J, Ferrer A, Conesa A. Differential expression in RNA-seq: a matter of depth. Genome research 2011;21(12):2213–2223.

26. Goldford JE, Lu N, Bajić D, Estrela S, Tikhonov M, Sanchez-Gorostiaga A, et al. Emergent simplicity in microbial community assembly. Science 2018;361(6401):469–474.
27. Merelli I, Pérez-Sánchez H, Gesing S, D'Agostino D. Managing, analysing, and integrating big data in medical bioinformatics: open problems and future perspectives. BioMed research international 2014;2014.
28. Sohn JI, Nam JW. The present and future of de novo whole-genome assembly. Briefings in Bioinformatics 2018 Jan;19(1):23–40.
29. Greene CS, Tan J, Ung M, Moore JH, Cheng C. Big data bioinformatics. Journal of cellular physiology 2014;229(12):1896–1900.
30. Pal S, Mondal S, Das G, Khatua S, Ghosh Z. Big data in biology: The hope and present-day challenges in it. Gene Reports 2020 Dec;21:100869. http://www.sciencedirect.com/science/article/pii/S2452014420302831.
31. Lampa S, Dahlö M, Olason PI, Hagberg J, Spjuth O. Lessons learned from implementing a national infrastructure in Sweden for storage and analysis of next-generation sequencing data. Gigascience 2013;2(1):2047-217X.
32. Sterling T, Brodowicz M, Anderson M. High performance computing: modern systems and practices. Morgan Kaufmann; 2017.
33. Wikipedia C, Supercomputing in Europe; 2021. [Online; accessed 2-April-2021]. https://en.wikipedia.org/w/index.php?title=Supercomputing_in_Europe&oldid=1009652575.
34. The Members of the PRACE Scientific Steering Committee. The scientific case for computing in Europe 2018 – 2026. ISBN: 9789082169492: Insight Publishers, Bristol, UK; 2018. https://prace-ri.eu/about/scientific-case/.
35. Candela L, Castelli D, Pagano P. Virtual research environments: an overview and a research agenda. Data Science Journal 2013;p. GRDI–013.
36. Haasjes GW, Containerization of legacy applications; 2021. https://developer.ibm.com/technologies/containers/articles/containerization-of-legacy-applications/.
37. Rad BB, Bhatti HJ, Ahmadi M. An introduction to docker and analysis of its performance. International Journal of Computer Science and Network Security (IJCSNS) 2017;17(3):228.
38. Kurtzer GM, Sochat V, Bauer MW. Singularity: Scientific containers for mobility of compute. PLOS ONE 2017;12(5):e0177459. https://journals.plos.org/plosone/article?id=10.1371/journal.pone.0177459.
39. Lagnel J, Manousaki T, Kotoulas G, Magoulas A. Biocluster: an NGS-dedicated HPC cluster in IMBBC, HCMR Upcoming Upgrade Usage/Partners Training. In: 9. Hellenic Bioinformatics 2016; 2016. .
40. Zafeiropoulos H, Gioti A, Ninidakis S, Potirakis A, Paragkamian S, Angelova N, et al. The IMBBC HPC facility: history and configuration, usage statistics, user management and task coordination data and related activities. Zenodo publication, preprint 2021;https://doi.org/10.5281/zenodo.4665308.
41. Dongarra JJ, Luszczek P, Petitet A. The LINPACK Benchmark: past, present and future. Concurrency and Computation: Practice and Experience 2003;15(9):803–820. https://onlinelibrary.wiley.com/doi/abs/10.1002/cpe.728, _eprint: https://onlinelibrary.wiley.com/doi/pdf/10.1002/cpe.728.
42. Castrignanò T, Gioiosa S, Flati T, Cestari M, Picardi E, Chiara M, et al. ELIXIR-IT HPC@CINECA: high performance computing resources for the bioinformatics community. BMC Bioinformatics 2020 Aug;21(10):352. https://doi.org/10.1186/s12859-020-03565-8.
43. Zafeiropoulos H, Viet HQ, Vasileiadou K, Potirakis A, Arvanitidis C, Topalis P, et al. PEMA: a flexible Pipeline for Environmental DNA Metabarcoding Analysis of the 16S/18S ribosomal RNA, ITS, and COI marker genes. GigaScience 2020 Mar;9(3):giaa022. https://academic.oup.com/gigascience/article/doi/10.1093/gigascience/giaa022/5803335.
44. Catchen J, Hohenlohe PA, Bassham S, Amores A, Cresko WA. Stacks: an analysis tool set for population genomics. Molecular Ecology 2013 Jun;22(11):3124–3140.
45. Varsos C, Patkos T, Oulas A, Pavloudi C, Gougousis A, Ijaz U, et al. Optimized R functions for analysis of ecological community data using the R virtual laboratory (RvLab). Biodiversity Data Journal 2016 Jan;4:e8357. https://bdj.pensoft.net/article/8357/.
46. Katsanevakis S, Coll M, Piroddi C, Steenbeek J, Ben Rais Lasram F, Zenetos A, et al. Invading the Mediterranean Sea: biodiversity patterns shaped by human activities. Frontiers in Marine Science 2014;1. https://www.frontiersin.org/articles/10.3389/fmars.2014.00032/full, publisher: Frontiers.
47. Klymus KE, Marshall NT, Stepien CA. Environmental DNA (eDNA) metabarcoding assays to detect invasive invertebrate species in the Great Lakes. PLOS ONE 2017;12(5):e0177643. https://journals.plos.org/plosone/article?id=10.1371/journal.pone.0177643, publisher: Public Library of Science.
48. Bariche M, Al-Mabruk SaA, Ateş MA, Büyük A, Crocetta F, Dritsas M, et al. New Alien Mediterranean Biodiversity Records (March 2020). Mediterranean Marine Science 2020 Apr;21(1):129–145. https://ejournals.epublishing.ekt.gr/index.php/hcmr-med-mar-sc/article/view/21987, number: 1.
49. Obst M, Exter K, Allcock AL, Arvanitidis C, Axberg A, Bustamante M, et al. A Marine Biodiversity Observation Network for Genetic Monitoring of Hard-Bottom Communities (ARMS-MBON). Frontiers in Marine Science 2020 Nov;7:572680. https://www.frontiersin.org/articles/10.3389/fmars.2020.572680/full.
50. Pauletto M, Manousaki T, Ferraresso S, Babbucci M, Tsakogiannis A, Louro B, et al. Genomic analysis of Sparus aurata reveals the evolutionary dynamics of sex-biased genes in a sequential hermaphrodite fish. Communications Biology 2018 Aug;1(1):1–13. https://www.nature.com/articles/s42003-018-0122-7.
51. Sarropoulou E, Sundaram AYM, Kaitetzidou E, Kotoulas G, Gilfillan GD, Papandroulakis N, et al. Full genome survey and dynamics of gene expression in the greater amberjack Seriola dumerili. GigaScience 2017 Dec;6(12). https://academic.oup.com/gigascience/article/6/12/gix108/4600083.
52. Tsakogiannis A, Manousaki T, Anagnostopoulou V, Stavroulaki M, Apostolaki ET. The Importance of Genomics for Deciphering the Invasion Success of the Seagrass Halophila stipulacea in the Changing Mediterranean Sea. Diversity 2020;12(7). https://www.mdpi.com/1424-2818/12/7/263.
53. Danis T, Tsakogiannis A, Kristoffersen JB, Golani D, Tsaparis D, Kasapidis P, et al. Building a high-quality reference genome assembly for the eastern Mediterranean Sea invasive sprinter Lagocephalus sceleratus (Tetraodontiformes, Tetraodontidae). bioRxiv 2020 Feb;p. 2020.02.17.952580. https://www.biorxiv.org/content/10.1101/2020.02.17.952580v2.
54. Angelova N, Danis T, Jacques L, Tsigenopoulos C, Manousaki T, SnakeCube: containerized and automated next- generation sequencing (NGS) pipelines for genome analyses in HPC environments. Zenodo; 2021. https://doi.org/10.5281/zenodo.4663112.
55. Natsidis P, Tsakogiannis A, Pavlidis P, Tsigenopoulos CS,

Manousaki T. Phylogenomics investigation of sparids (Teleostei: Spariformes) using high-quality proteomes highlights the importance of taxon sampling. Communications Biology 2019 Nov;2(1):1–10. https://www.nature.com/articles/s42003-019-0654-5, number: 1 Publisher: Nature Publishing Group.

56. Sarropoulou E, Sepulcre P, Poisa-Beiro L, Mulero V, Meseguer J, Figueras A, et al. Profiling of infection specific mRNA transcripts of the European seabass Dicentrarchus labrax. BMC Genomics 2009 Apr;10(1):157. https://doi.org/10.1186/1471-2164-10-157.
57. Papadaki M, Kaitetzidou E, Mylonas CC, Sarropoulou E. Non-coding RNA Expression Patterns of Two Different Teleost Gonad Maturation Stages. Marine Biotechnology 2020;22(5):683–695.
58. Warwick RM, Somerfield PJ. All animals are equal, but some animals are more equal than others. Journal of Experimental Marine Biology and Ecology 2008 Nov;366(1):184–186. http://www.sciencedirect.com/science/article/pii/S0022098108003444.
59. Arvanitidis CD, Warwick RM, Somerfield PJ, Pavloudi C, Pafilis E, Oulas A, et al. Research Infrastructures offer capacity to address scientific questions never attempted before: Are all taxa equal? PeerJ Inc.; 2018.
60. Vandepitte L, Vanhoorne B, Decock W, Vranken S, Lanssens T, Dekeyzer S, et al. A decade of the World Register of Marine Species – General insights and experiences from the Data Management Team: Where are we, what have we learned and how can we continue? PLOS ONE 2018;13(4):e0194599. https://journals.plos.org/plosone/article?id=10.1371/journal.pone.0194599, publisher: Public Library of Science.
61. Gioti A, Siaperas R, Nikolaivits E, Goff GL, Ouazzani J, Kotoulas G, et al. Draft Genome Sequence of a Cladosporium Species Isolated from the Mesophotic Ascidian Didemnum maculosum. Microbiology Resource Announcements 2020 Apr;9(18). https://mra.asm.org/content/9/18/e00311-20, publisher: American Society for Microbiology Section: Genome Sequences.
62. Nikolaivits E, Siaperas R, Agrafiotis A, Ouazzani J, Magoulas A, Gioti A, et al. Functional and transcriptomic investigation of laccase activity in the presence of PCB29 identifies two novel enzymes and the multicopper oxidase repertoire of a marine-derived fungus. Science of The Total Environment 2021 Jun;775:145818. https://www.sciencedirect.com/science/article/pii/S0048969721008858.
63. Dagum L, Menon R. OpenMP: an industry standard API for shared-memory programming. IEEE Computational Science and Engineering 1998 Jan;5(1):46–55. Conference Name: IEEE Computational Science and Engineering.
64. Vouzis PD, Sahinidis NV. GPU-BLAST: using graphics processors to accelerate protein sequence alignment. Bioinformatics 2011 Jan;27(2):182–188. https://doi.org/10.1093/bioinformatics/btq644.
65. Nobile MS, Cazzaniga P, Tangherloni A, Besozzi D. Graphics processing units in bioinformatics, computational biology and systems biology. Briefings in Bioinformatics 2017 Sep;18(5):870–885. https://doi.org/10.1093/bib/bbw058.
66. Mell P, Grance T, et al., The NIST definition of cloud computing. Computer Security Division, Information Technology Laboratory; 2011.
67. Langmead B, Nellore A. Cloud computing for genomic data analysis and collaboration. Nature Reviews Genetics 2018;19(4):208.
68. Dahlö M, Scofield DG, Schaal W, Spjuth O. Tracking the NGS revolution: managing life science research on shared high-performance computing clusters. GigaScience 2018 May;7(giy028). https://doi.org/10.1093/gigascience/giy028.

Figure1: imbbc hpc history

Click here to access/download;Figure;imbbc_hpc_timeline.png

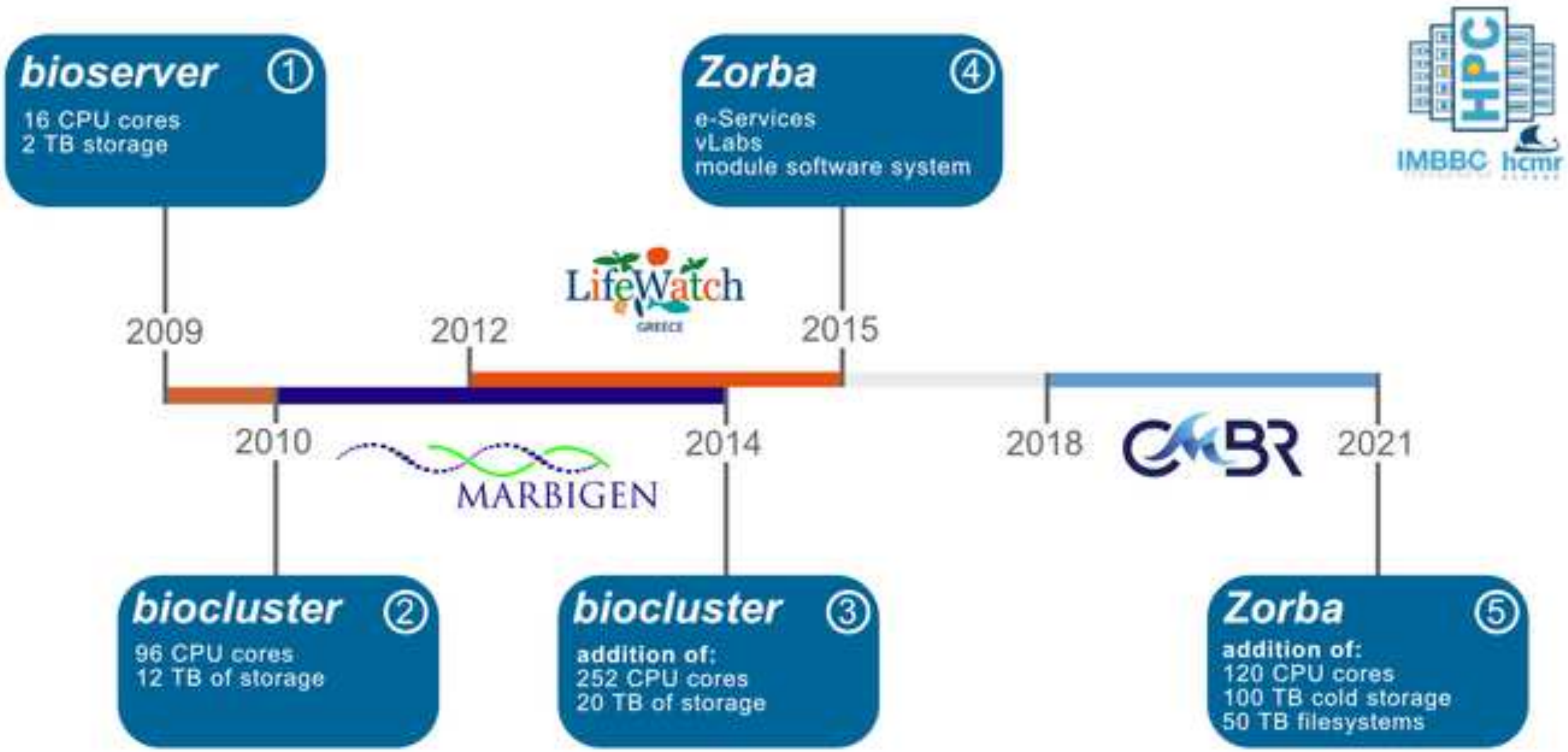

Figure2: zorba configuration

Click here to access/download;Figure;zorba_architecture.png

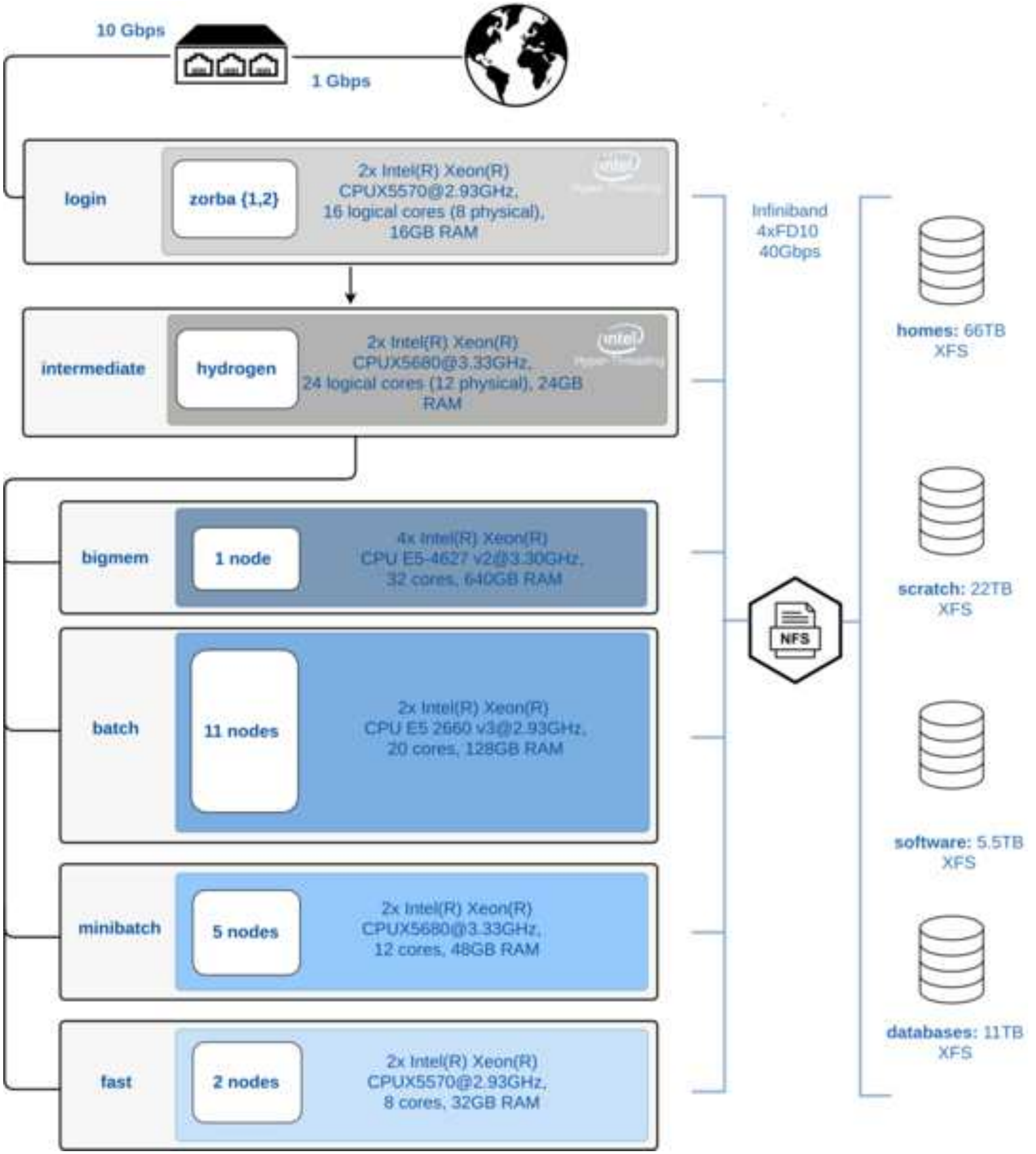

Figure3: studies per field

Click here to access/download;Figure;number_of_studies_biological_fields.pn

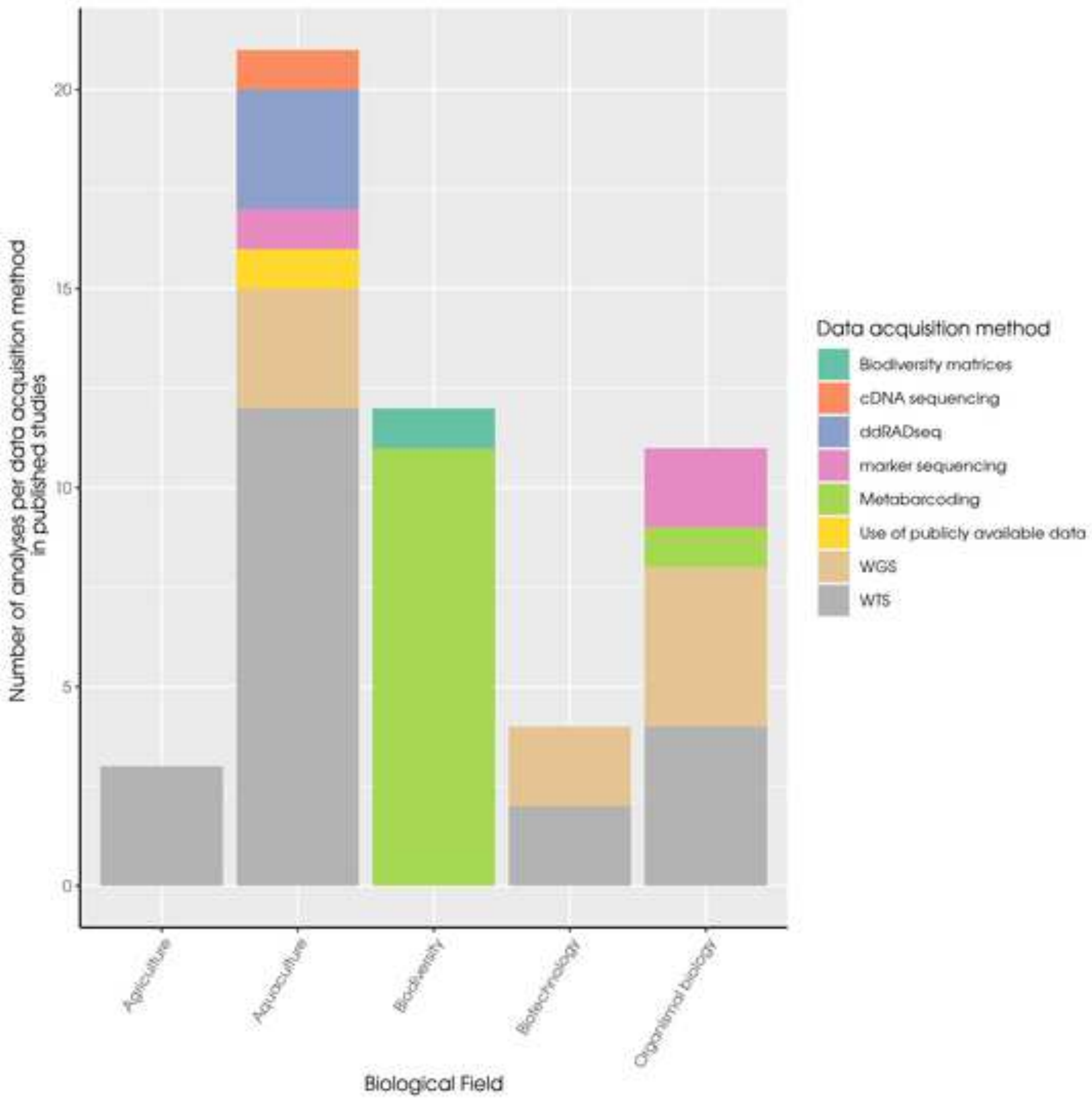

Figure4: computational requirements

Click here to access/download;Figure;computational_method_computational_requirement_plot.pn

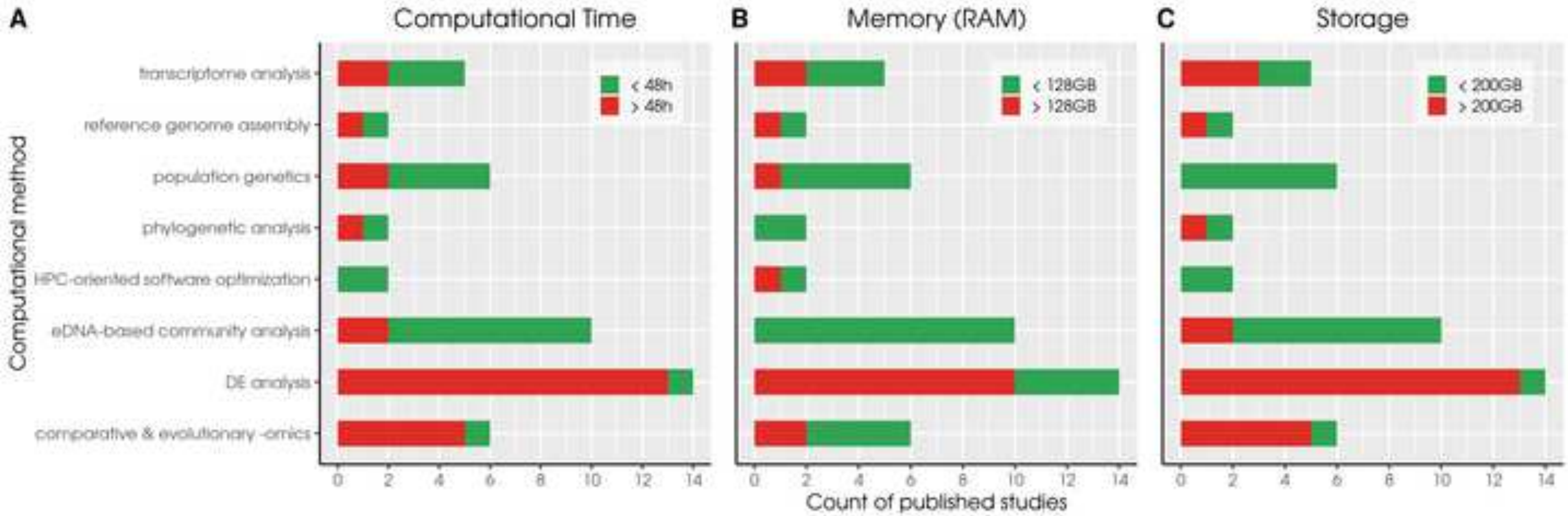

Supplement: giab053_GIGA-D-21-00111_Revision_1 [file giab053_giga-d-21-00111_revision_1.pdf]
