# Supplementary material for: 0s and 1s in marine molecular research: a regional HPC perspective
Source: Gigascience. 2021 Aug 18;10(8):giab053. doi: 10.1093/gigascience/giab053 (PMC8371273; doi:10.1093/gigascience/giab053)
Supplement: giab053_Response_to_Reviewer_Comments_Original_Submission [file giab053_response_to_reviewer_comments_original_submission.pdf]

Author's Response To Reviewer Comments

Close

We would like to kindly thank the reviewers for the time they spent to thoroughly read our manuscript. We appreciate all their accurate comments, which helped at the improvement of our manuscript. In the revised version, we have addressed all of the reviewer's comments and suggestions and have incorporated changes/amendments to the manuscript (indicated in cyan), where necessary. Below we cite our detailed answers to the editor and reviewers' comments and suggestions (reviewer text starts with a hash #).

Reviewer reports:
The paper is a retrospective on 12 years of running what has become a regional HPC facility in Greece. It reviews # the gradual evolution of a single-server resource into a Tier-2 facility, gives some insights into how the facility is
organized and run, highlights some of the research done using the facility, and offers some lessons learned from # these 12 years. In this, it should be of interest to researchers who use HPC facilities and those who run them.
The paper is well written and well organized, making it very accessible even to non-specialists. There are a
number of observations that I would make with a view to improving the relevance of the paper, and a minor
correction for readability.

Reviewer #1 comment #1:
1. Containerization: This is perhaps one of the more important recent developments in the HPC space (and
beyond). It is treated in a number of sections where, if I understand correctly, Singularity is the adopted
platform. #But what is the standard image format used? Singularity for direct compatibility with the HPC
environment, or #Docker for eventual external compatibility (e.g. with cloud compute)? What reasoning led to
any decisions here.

We chose Singularity as the standard image format for Zorba, while In terms of locally-developed containers, we support both technologies. Singularity was preferred because it ensures compatibility with our job scheduling system (i.e. SLURM). Singularity has been developed to perform complex applications on HPC environments focusing specifically on the needs of the scientific community and aiming to support reproducible science in a simple way. Therefore, it has a completely different approach compared to Docker regarding the namespace (allowing to setup a container without privileged operations, so that a non-root user can act as root inside a container without being root on the host outside) and filesystems, and supports Message Passing Interface (MPI). Docker, on the other hand, is not designed for data processing, but for running microservices (e.g. web-services). Moreover, Singularity is compatible with Docker containers, allowing to take advantage of all the benefits of Docker; e.g the large number of already developed containers and its larger community. Thus, Singularity is commonly used by Academic and Research institutions as the containerization technology for their HPC-oriented containers [1].
We have clarified that we use Singularity in the corresponding section (lines 157-158). While Singularity is our container technology of choice regarding running containers in our HPC facility, we also employ Docker for external compatibility purposes, i.e. the containers we create and share. The newly added last paragraph of Section "5D. The way forward: Develop locally, share and deploy centrally" and section "6. Cloud Computing" elaborate on such Docker use and its synergy with Cloud/HPC systems.

Reference
[1] Kurtzer, Gregory M., Vanessa Sochat, and Michael W. Bauer. "Singularity: Scientific containers for mobility of compute." PloS one 12.5 (2017): e0177459.

It would be very interesting to hear the authors' opinion on what developments they expect, or hope for, with
regards to containerization.

Containers are an already established technology, some of the largest cloud providers worldwide (e.g. Google Cloud, Azure, Amazon Web Services) have already adopted them to a great extent. Container use is also on the rise in research; it is our belief that it will be further accelerated in the future, especially in the context of FAIRification efforts. Despite "indirect costs" related to these efforts, such as costs to containerize legacy software, we believe that these technologies will become the norm. Ongoing, community-driven efforts, such as the BioContainers project (https://biocontainers.pro/) that provides both the infrastructure and the basic guidelines to build, manage and distribute images and containers, play a great part towards this direction. Additional contributions in the same direction are provided by a number of groups, institutes and universities that have already started to containerize and distribute already-developed and commonly known bioinformatics software tools (e.g the Hurwitz Lab container repository (https://github.com/hurwitzlab?q=singularity+OR+docker)). We have added a comment on our view regarding the future of containers at the end of section "5D. The way forward: Develop locally, share and deploy centrally" (lines 530 - 545).

Reviewer #1 comment #2:
2. Cloud computing: Commercial cloud providers and the potential for hybridization with traditional "in-house"
HPC, is another topic that is relevant today for research that requires significant computation. This was touched
on very briefly in the first of the Lessons Learned sub-sections, where budget and containerization were
mentioned. I suspect that other HPC managers and staff would welcome some more detail here. What do the
authors consider to be the factors that would lead towards the (increased) use of cloud computing? What are
the drawbacks? Had any comparison of costs been undertaken? Are there any mismatches between the HPC
model of containerization, and that of commercial cloud providers? (e.g. Docker vs Singularity) that would need # to be addressed?

There is a trend for cloud computing services managed by a web interface. This is mainly because they offer simplicity and high availability to users with less or even no experience in HPC systems. In addition, the time needed for data manipulation, software installation and user-system interaction is significantly reduced compared to a local HPC facility. On the other hand, tool experimentation and benchmarking is more limited in cloud computing compared to local facilities, which are in general more dedicated to specific research areas. While a cost comparison e.g. among the reported HPC usage and equivalent solutions in the cloud is possible, a thorough one has not been conducted. Since our facility still relies on legacy (i.e. not containerized) software and workflows, we are in a preparatory phase towards an era where cloud resources can be optimally used, and thus an exhaustive cost comparison is not relevant at this stage for Zorba. The more progress is made, the more such a strategic shift would make sense in the future. In addition, non-profit, academia-oriented, cloud computing solutions will be explored first prior to commercial ones. Regarding mismatches between the two systems on containerization: Although both fully support Singularity containers, Singularity has been specifically developed to support HPC systems, while Docker is designed to run in isolated environments (i.e. Virtual machines), as explained in comment 1. As elaborated further in comment 4 and by the means of comprehensiveness, a paragraph on "6. Cloud computing" has been added in the manuscript.

Reviewer #1 comment #3:
3. Multi-threaded programming: The authors correctly mention the use of software threads as the general
solution to parallelization in bioinformatics programs. But there is a problem in the way that they characterize

multi-node parallelization. The authors point out that to distribute parallel tasks over multiple nodes, threading
is not enough. The typical solution for bioinformatic programmers is to use MPI (across nodes) alongside
OpenMP (within nodes). The authors point out that this is only used in a small minority of bioinformatic
applications, and that MPI usage is low in others. As a reviewer, I presume that this is an indication, from the
authors, that cross-node parallelization (i.e. fully distributed) is not something that happens often on their
facility, or in any case is considered to be niche enough to influence the choice of node types on the cluster
(presumably in favour of nodes with large numbers of cores). Firstly, could I ask the authors if I have correctly
interpreted the message of the subsection entitled "Software optimizations for parallel execution"?

The reviewer has correctly understood that cross-node parallelization does not happen often on our facility, mainly because there is a limited number of tools that exploit similarly well cores from multiple nodes (multi-node parallelization). This has indeed partially influenced our strategic choices on the system architecture.

If so, I would like to offer another perspective on the matter, and would welcome the authors' thoughts. OpenMP
and MPI programming is difficult. They are both low-level abstractions that force the coder to concentrate on
implementation details close to the OS and hardware. I believe it is *this* difficulty that leads to this approach
being used only in niche settings, and that if easier approaches to multi-node distribution were available,
bioinformaticians would make use of them. Such approaches do exist (actor-based distribution as exemplified by
the Akka library is one) but they are not generally known to bioinformaticians. They offer high-level abstractions
that hide the details of both intra-node threading and inter-node Inter Process Communication. If
bioinformaticians were introduced to these techniques, it is possible that they could make more efficient use of
HPC (and/or cloud) infrastructure.

We agree that MPI and (in some cases when numerous dependencies exist) OpenMP require more in-depth knowledge and experience in how to capitalize on multi-core systems (mostly MPI, since OpenMP is less complex). Actor-based parallel model is indeed a more understandable way of producing concurrency and could become popular among bioinformaticians. We thank the reviewer for the suggestion; it could be further explored once a pertinent IMBBC HPC use case arises.

I think this is a chicken-and-egg situation, where users of HPC facilities are encouraged to use MPI/OpenMP,
and HPC facilities operate as if these low-level protocols were the only available approach. The authors mention
"training as an integral component to the HPC mindset". Do they see an opportunity here to use that training to
extend the computational reach of bioinformaticians?

The vast majority of Zorba users make use of third-party software tools (not developing their own) for their workflows, thus the Zorba team’s primary care so far has been to render users more familiar with how different parallel technologies work, so as to understand how to use these more efficiently in terms of hardware resources. Once efficient and user-friendly models for parallelization become widely acceptable in the community, we do foresee adopting them and training Zorba users in these. In addition, The Zorba team and many researchers in the Institute actively follow related training activities (e.g. by ELIXIR, EOSC), which are commonly based on user surveys to identify current needs and future goals in terms of computing.

#Reviewer #1 comment #4:
#4. Network intensive processing: The first Lessons Learned subsection explores the extent to which processes are
memory, or CPU bound. No mention is made of network intensity. In a way this point extends from the previous
one about fully-distributed processes, and it also touches on the cloud vs HPC question. It might be said that the
distinguishing feature of a HPC facility (over say a private or public cloud) is the InfiniBand network layer. So the
question of making full use of this feature is relevant. I'd welcome some discussion on the general point of HPC
# vs cloud in the paper, and about InfiniBand's role in that comparison, especially in the section that looks to the
future of the facility.

A large number of bioinformatics tools and housekeeping software running on Zorba are network-intensive, either due to I/O operations they perform in shared filesystems, or to node intercommunication. To address this issue we adopted IB, as mentioned in the companion preprint paper (https://zenodo.org/record/4665308, see section “A2. The Zorba configuration of the IMBBC HPC facility” therein). The reviewer is right that we did not mention network intensity in the main text. Since it is indeed important for the discussion, we have added relevant information in the revised manuscript, lines 132 - 137.
Infiniband (IB) is the key element to efficiently serve intercommunication among structural parts of an HPC facility (either traditional or cloud-based); therefore, investment in high bandwidth (based on RDMA technology) is important. In practise, processes using MPI are those that really take advantage of IB, mainly in traditional HPC systems when running MPI among a large number of nodes. In cloud infrastructures, IB is also important in cases when resources from different nodes are combined to provide a unified virtual machine. However, most commercial Cloud providers do not support IB interconnection. In that case, fast direct memory access between the nodes is needed in order to ensure a similar or equal result in terms of performance, as if it ran on a single server. However, IB cannot really accelerate IO operations (e.g. file transfers, data reads and writes) since it is bounded by the filesystem type, the disks and the concurrent demand at a specific moment. Some of the above points are mentioned in our revised manuscript, new paragraph “6. Cloud computing”.

#Reviewer #1 comment #5:
5. Minor point - the paper has references to numbered sections (e.g. "Section 2") but there is no numbering in
the paper format.

Section numbering has been updated.

#Reviewer #2 comment #1:
The manuscript presents a description and importance of High-performance computing (HPC) in marine
research. The topic is of interest because allows important studies in marine research. The manuscript describes
the system architecture and capacity of processing of Zorba facility. I think that the important point of the Zorba
facility is concern a accessible communication between users and administrators. The method is quite standard
and is appropriate for the study, especially given that the main focus of the paper is to describe the evolution of
IMBBC HPC facility during the 12 past years. Is notable the progress of the facility in the analysis of a wide
variety of information. The conclusions are satisfactory. The solutions applied in hardware (depth/breadth
balanced structure, user quotas and temporary storage), software (modularized bioinformatics

application
maintenance and containerization are very interesting) and training enable that the scientific community can
deal with a wide # variety of information. The language is clear and does not require a heavy edition and the
statistical methods are not relevant for the present paper.

We would like to thank the reviewer for the positive comments.

#Reviewer #2 comment #2:
Minor revisions
In the topic "Computational breakdown of the IMBBC HPC supported research", please fix the order of the
figures. It seems to me that is Figure 4 and not Figure 3. In the phrases "As shown in Fig. 3...", "Long
computational times (Fig 3A)", "... approaches is the significant storage limitations (Fig 3C)..." Figure 4 is not
clearly labeled, please see the text of 4B and 4C.

All mismatched annotations have been fixed in the revised manuscript.

Reviewer #3 comment #1:
The manuscript provides an accessible and informative review of the evolving needs and solutions for High
Performance Computing at a regional scale serving users in the broad domain of marine science and
biotechnology. For readers without deep expertise in computational science (as is the case for this reviewer) the
authors are commended in providing a useful insight to the challenges facing such cyberinfrastructure and how
those challenges have been addressed in the past as well as some future guidance. The use cases provided were
appropriate and illustrative of the types of study supported by this HPC facility, and the heterogenous demands
for types of computing resource and how these can be met and balanced among the needs of different users.
The common challenge of storage was well expounded but solutions appear to be still rather elusive.

We would like to thank the reviewer for the positive comments. We agree that storage is a major challenge and is not easy to be met. A storage upgrade scheduled within 2021 (see Section “7. Future Directions”) is expected to alleviate current storage challenges in Zorba. However, given the ever increasing data production (e.g. as the result of decreasing sequencing costs, and/or of rising imaging technologies), responsible storage use approaches as described here remain only partial solutions to anticipated future storage needs. Centralized (Tier-1 or higher) storage solutions represent a longer-term solution, which is in line with current views on how to handle big data generated by international research consortia in a long-lasting manner.
We have added the above comments in the revised manuscript, in section 5B “Quota... overloaded” lines 465 - 475.

Close
