# Supplementary material for: 0s and 1s in marine molecular research: a regional HPC perspective
Source: Gigascience. 2021 Aug 18;10(8):giab053. doi: 10.1093/gigascience/giab053 (PMC8371273; doi:10.1093/gigascience/giab053)
Supplement: giab053_Reviewer_1_Report_Original_Submission — Brendan Lawlor -- 5/1/2021 Reviewed [file giab053_reviewer_1_report_original_submission.pdf]

Reviewer Report

Title: 0s and 1s in marine molecular research: a regional HPC perspective

Version: Original Submission **Date:** 5/1/2021

Reviewer name: Brendan Lawlor

Reviewer Comments to Author:

The paper is a retrospective on 12 years of running what has become a regional HPC facility in Greece. It reviews the gradual evolution of a single-server resource into a Tier-2 facility, gives some insights into how the facility is organized and run, highlights some of the research done using the facility, and offers some lessons learned from these 12 years. In this, it should be of interest to researchers who use HPC facilities and those who run them.

The paper is well written and well organized, making it very accessible even to non-specialists.

There are a number of observations that I would make with a view to improving the relevance of the paper, and a minor correction for readability.

1. Containerization: This is perhaps one of the more important recent developments in the HPC space (and beyond). It is treated in a number of sections where, if I understand correctly, Singularity is the adopted platform. But what is the standard image format used? Singularity for direct compatibility with the HPC environment, or Docker for eventual external compatibility (e.g. with cloud compute)? What reasoning led to any decisions here. It would be very interesting to hear the authors' opinion on what developments they expect, or hope for, with regards to containerization.

2. Cloud computing: Commercial cloud providers and the potential for hybridization with traditional "in-house" HPC, is another topic that is relevant today for research that requires significant computation. This was touched on very briefly in the first of the Lessons Learned sub-sections, where budget and containerization were mentioned. I suspect that other HPC managers and staff would welcome some more detail here. What do the authors consider to be the factors that would lead towards the (increased) use of cloud computing? What are the drawbacks? Had any comparison of costs been undertaken? Are there any mismatches between the HPC model of containerization, and that of commercial cloud providers? (e.g. Docker vs Singularity) that would need to be addressed?

3. Multi-threaded programming: The authors correctly mention the use of software threads as the general solution to parallelization in bioinformatics programs. But there is an problem in the way that they characterize multi-node parallelization. The authors point out that to distribute parallel tasks over multiple nodes, threading is not enough. The typical solution for bioinformatic programmers is to use MPI (across nodes) alongside OpenMP (within nodes). The authors point out that this is only used in a small minority of bioinformatic applications, and that MPI usage is low in others. As a reviewer, I presume that this is an indication, from the authors, that cross-node parallelization (i.e. fully distributed) is not something that happens often on their facility, or in any case is considered to be niche enough to influence the choice of node types on the cluster (presumably in favour of nodes with large numbers of cores). Firstly, could I ask the authors if I have correctly interpreted the message of the subsection entitled "Software optimizations for parallel execution"? If so, I would like to offer another perspective

on the matter, and would welcome the authors' thoughts. OpenMP and MPI programming is difficult. They are both low-level abstractions that force the coder to concentrate on implementation details close to the OS and hardware. I believe it is *this* difficulty that leads to this approach being used only in niche settings, and that if easier approaches to multi-node distribution were available, bioinformaticians would make use of them. Such approaches do exist (actor-based distribution as exemplified by the Akka library is one) but they are not generally known to bioinformaticians. They offer high-level abstractions that hide the details of both intra-node threading and inter-node Inter Process Communication. If bioinformaticians were introduced to these techniques, it is possible that they could make more efficient use of HPC (and/or cloud) infrastructure. I think this is a chicken-and-egg situation, where users of HPC facilities are encouraged to use MPI/OpenMP, and HPC facilities operate as if these low-level protocols were the only available approach. The authors mention "training as an integral component to the HPC mindset". Do they see an opportunity here to use that training to extend the computational reach of bioinformaticians?
4. Network intensive processing: The first Lessons Learned subsection explores the extent to which processes are memory, or CPU bound. No mention is made of network intensity. In a way this point extends from the previous one about fully-distributed processes, and it also touches on the cloud vs HPC question. It might be said that the distinguishing feature of a HPC facility (over say a private or public cloud) is the InfiniBand network layer. So the question of making full use of this feature is relevant. I'd welcome some discussion on the general point of HPC vs cloud in the paper, and about InfiniBand's role in that comparison, especially in the section that looks to the future of the facility.
5. Minor point - the paper has references to numbered sections (e.g. "Section 2") but there is no numbering in the paper format.

Methods

Are the methods appropriate to the aims of the study, are they well described, and are necessary controls included? Choose an item.

Conclusions

Are the conclusions adequately supported by the data shown? Choose an item.

Reporting Standards

Does the manuscript adhere to the journal's guidelines on [minimum standards of reporting?](#) Choose an item.

Choose an item.

Statistics

Are you able to assess all statistics in the manuscript, including the appropriateness of statistical tests used? Choose an item.

Quality of Written English

Please indicate the quality of language in the manuscript: Choose an item.

Declaration of Competing Interests

Please complete a declaration of competing interests, considering the following questions:

- Have you in the past five years received reimbursements, fees, funding, or salary from an organisation that may in any way gain or lose financially from the publication of this manuscript, either now or in the future?
- Do you hold any stocks or shares in an organisation that may in any way gain or lose financially from the publication of this manuscript, either now or in the future?
- Do you hold or are you currently applying for any patents relating to the content of the manuscript?
- Have you received reimbursements, fees, funding, or salary from an organization that holds or has applied for patents relating to the content of the manuscript?
- Do you have any other financial competing interests?
- Do you have any non-financial competing interests in relation to this paper?

If you can answer no to all of the above, write 'I declare that I have no competing interests' below. If your reply is yes to any, please give details below.

I declare that I have no competing interests

I agree to the open peer review policy of the journal. I understand that my name will be included on my report to the authors and, if the manuscript is accepted for publication, my named report including any attachments I upload will be posted on the website along with the authors' responses. I agree for my report to be made available under an Open Access Creative Commons CC-BY license (http://creativecommons.org/licenses/by/4.0/). I understand that any comments which I do not wish to be included in my named report can be included as confidential comments to the editors, which will not be published.

Choose an item.

To further support our reviewers, we have joined with Publons, where you can gain additional credit to further highlight your hard work (see: https://publons.com/journal/530/gigascience). On publication of this paper, your review will be automatically added to Publons, you can then choose whether or not to claim your Publons credit. I understand this statement.

Yes Choose an item.
