# Supplementary material for: 0s and 1s in marine molecular research: a regional HPC perspective
Source: Gigascience. 2021 Aug 18;10(8):giab053. doi: 10.1093/gigascience/giab053 (PMC8371273; doi:10.1093/gigascience/giab053)
Supplement: giab053_Reviewer_2_Report_Original_Submission — Graciela Dias -- 5/22/2021 Reviewed [file giab053_reviewer_2_report_original_submission.pdf]

Reviewer Report

Title: 0s and 1s in marine molecular research: a regional HPC perspective

Version: Original Submission **Date:** 5/22/2021

Reviewer name: Graciela Dias

Reviewer Comments to Author:

The manuscript presents a description and importance of High-performance computing (HPC) in marine research. The topic is of interest because allows important studies in marine research. The manuscript describes the system architecture and capacity of processing of Zorba facility. I think that the important point of the Zorba facility is concern a accessible communication between users and administrators.
The method is quite standard and is appropriate for the study, especially given that the main focus of the paper is to describe the evolution of IMBBC HPC facility during the 12 past years. Is notable the progress of the facility in the analysis of a wide variety of information.
The conclusions are satisfactory. The solutions applied in hardware (depth/breadth balanced structure, user quotas and temporary storage), software (modularized bioinformatics application maintenance and containerization are very interesting) and training enable that the scientific community can deal with a wide variety of information.
The language is clear and does not require a heavy edition and the statistical methods are not relevant for the present paper.
Minor revisions
In the topic "Computational breakdown of the IMBBC HPC supported research", please fix the order of the figures. It seems to me that is Figure 4 and not Figure 3. In the phrases "As shown in Fig. 3...", "Long computational times (Fig 3A)", "... approaches is the significant storage limitations (Fig 3C)..."
Figure 4 is not clearly labeled, please see the text of 4B and 4C.

Methods

Are the methods appropriate to the aims of the study, are they well described, and are necessary controls included? Choose an item.

Conclusions

Are the conclusions adequately supported by the data shown? Choose an item.

Reporting Standards

Does the manuscript adhere to the journal's guidelines on minimum standards of reporting? Choose an item.

Choose an item.

Statistics

Are you able to assess all statistics in the manuscript, including the appropriateness of statistical tests used? Choose an item.

Quality of Written English

Please indicate the quality of language in the manuscript: Choose an item.

Declaration of Competing Interests

Please complete a declaration of competing interests, considering the following questions:

- Have you in the past five years received reimbursements, fees, funding, or salary from an organisation that may in any way gain or lose financially from the publication of this manuscript, either now or in the future?
- Do you hold any stocks or shares in an organisation that may in any way gain or lose financially from the publication of this manuscript, either now or in the future?
- Do you hold or are you currently applying for any patents relating to the content of the manuscript?
- Have you received reimbursements, fees, funding, or salary from an organization that holds or has applied for patents relating to the content of the manuscript?
- Do you have any other financial competing interests?
- Do you have any non-financial competing interests in relation to this paper?

If you can answer no to all of the above, write 'I declare that I have no competing interests' below. If your reply is yes to any, please give details below.

I declare that I have no competing interests

I agree to the open peer review policy of the journal. I understand that my name will be included on my report to the authors and, if the manuscript is accepted for publication, my named report including any attachments I upload will be posted on the website along with the authors' responses. I agree for my report to be made available under an Open Access Creative Commons CC-BY license (http://creativecommons.org/licenses/by/4.0/). I understand that any comments which I do not wish to be included in my named report can be included as confidential comments to the editors, which will not be published.

Choose an item.

To further support our reviewers, we have joined with Publons, where you can gain additional credit to further highlight your hard work (see: https://publons.com/journal/530/gigascience). On publication of this paper, your review will be automatically added to Publons, you can then choose whether or not to claim your Publons credit. I understand this statement.

Yes Choose an item.
